# Assessing potential impacts of the EVFTA on Vietnam’s pharmaceutical imports from the EU: an application of SMART analysis

**DOI:** 10.1186/s40064-016-3200-7

**Published:** 2016-09-07

**Authors:** Huong Thanh Vu

**Affiliations:** Faculty of International Business and Economics, University of Economics and Business, Vietnam National University – Hanoi, 144 Xuan Thuy, Cau Giay, Hanoi, Vietnam

**Keywords:** EVFTA, Vietnam, Pharmaceuticals, SMART, TPP, ASEAN + 3

## Abstract

This paper by adopting the Software on Market Analysis and Restrictions on Trade assessed the ex-ante impact of tariff elimination under the European—Vietnam free trade agreement (EVFTA) on Vietnam’s pharmaceutical imports from the EU based on two scenarios. The results showed that although Vietnam’s tariff removal for the EU’s medicines would not result in a significant increase in Vietnam’s imports from the EU, Vietnam’s deeper integration with ASEAN + 3 and TPP (the Trans-Pacific Partnership) nations would affect quite slightly on its imports from the EU. Therefore, the EU would be still the most important and biggest source of pharmaceuticals for Vietnam in the near future. In addition, there might be an uneven distribution in Vietnam’s import increases by the EU nation, pharmaceutical group and product. The simulation results also pointed out that the EVFTA’s trade creation effect would be higher than trade diversion effect and therefore the agreement would improve welfare of Vietnam. When Vietnam extends its coverage of tariff elimination to also TPP and ASEAN + 3, Vietnam’s welfare would potentially increase more but Vietnam would face with the relatively high increases of pharmaceutical imports from not only the EU but also the US, Australia, South Korea, Thailand and China. Bases on these results, the paper argued that both the Vietnamese government and pharmaceutical enterprises should not neglect the EVFTA and its impacts on the pharmaceutical sector, and perceive clearly the uneven distribution of Vietnam’s import changes from the EU by nation and by product to design appropriate business and investment strategy. In addition, Vietnam should take measures to diversify its European import markets to be less dependent on the traditional ones in the current context of the EU. Finally, Vietnam should promote the integration in the pharmaceutical sector with all three groups of nations, especially ASEAN and ASEAN’s key partners, to reduce trade diversion effect and raise the welfare of Vietnam, given that Vietnam should consider carefully the point of time to remove tariff for each group to avoid the sudden increase in its pharmaceutical imports.

**Electronic supplementary material:**

The online version of this article (doi:10.1186/s40064-016-3200-7) contains supplementary material, which is available to authorized users.

## Background

Being a tropical monsoon climate country, a great deal of diseases are more likely to be easily occurred in Vietnam, whose population ranks 14th in the world (CIA 2014) and population density is high among the South East Asian nations (EVBN 2014). Vietnam is also facing with the risk of environmental pollution and lack of safety in the daily food. However, in the recent years, Vietnam’s living standard, awareness of healthcare issues and the access to medicines have been improved. All of the above factors have resulted in an increasing demand for medicines in Vietnam and made Vietnam become a highly lucrative pharmaceutical market with the highest growth rate among the South East Asian nations (Vu 2014).

The pharmaceutical market of Vietnam has been characterized by imported drugs, which have meet nearly 60 % of the total domestic consumption in the recent years (Nguyen 2014b; Maybank KimEng 2015). Among the pharmaceutical import markets of Vietnam, the European Union (EU) has traditionally been the largest one. Overtime, imports from the EU of Vietnam have continuously increased and reached USD 1.1 billion, accounting for nearly 51 % of Vietnam’s total pharmaceutical imports in 2014 (ITC 2016). Pharmaceuticals are also the second biggest products Vietnam imported from the EU during the period 2001–2014 and have significantly contributed to meet the domestic demand, take care of the people’s health and stabilize the socio-economic development of Vietnam.

On 2nd December 2015, Vietnam and the EU signed the Declaration on the conclusion of the European—Vietnam Free Trade Agreement (EVFTA) negotiation and on 1st February 2016, the full text of the agreement was officially announced. The way ahead now for both parties is to conduct legal review, translate the EVFTA into the EU’s official languages and Vietnamese, approve and ratify the agreement. According to information published so far, Vietnam commits to eliminate tariff for about a half of pharmaceuticals’ tariff lines immediately on the date EVFTA enters into force and the rest shall be removed within 10 years (Vu 2015). As the EU is Vietnam’s largest pharmaceutical import market, this tariff elimination is likely to affect considerably Vietnam’s pharmaceutical imports and healthcare industry. Therefore, understanding the impact of tariff removal under the EVFTA on Vietnam’s pharmaceutical imports is vital for both the Vietnamese government and enterprises, contributing to support them to better and more efficiently prepare for integration with the EU and avoid adverse effects on the development of this industry.

In Vietnam, while the impact of TPP (the Trans-Pacific Partnership) on Vietnam’s economy is a common topic, the impact of EVFTA is neglected by both researchers and enterprises. So far, there are just some comprehensive research on the impact of the EVFTA on Vietnam’s economy such as those conducted by Philip et al. (2011), Baker et al. (2014), and Nguyen (2014a). More importantly, there is no previous literature quantifying the impact of the EVFTA on Vietnam’s trade in pharmaceuticals while this product is among the top traded goods between Vietnam and the EU, and the EU is the biggest source supplying this product to Vietnam. To fill this gap, this paper, by adopting the Software on Market Analysis and Restrictions on Trade (SMART), helps answer how the EVFTA potentially affects Vietnam’s imports of pharmaceuticals from the EU. The paper is structured as below. After the introduction, the second part reviews some past literature, and the next two parts analyze key features in Vietnam’s pharmaceutical imports from the EU and tariff reduction commitments under the EVFTA. The methodology and data are presented in the fifth part and then results on potential impacts of the EVFTA are shown in the sixth part. The paper draws out some implications, which are essential for Vietnam to better prepare for as well as take advantages of this upcoming ambitious free trade agreement (FTA) in the final part before coming up with the conclusions. The pioneering study in Vietnam adopting the SMART to examine and quantify the impacts of the EVFTA on Vietnam’s imports of pharmaceuticals from the EU is the biggest contribution of this paper.

## Literature review

### Literature review on trade impacts of a FTA

In the context of Doha Round failure, FTAs are considered the second best option for nations to promote international economic integration. Together with the strong development of FTAs all over the world, and wider and deeper coverage of FTA negotiations, the contents of a new-generation FTA are now not limited to trade liberalization but also extends to other more complicated issues such as investment, government procurement, intellectual property right, environment and labor (Matsushita 2010; VCCI et al. 2012). However, the key background and foremost objectives of a FTA, especially a FTA involved developing countries, are so far still trade liberalization and therefore the trade impacts of a FTA have been key attention of both government and enterprises in the developing countries.

Trade impacts of a FTA have widely been accepted among scholars to include static and dynamic effects. Analysis of static impacts is often based on the theory of customs union and influenced by Viner (1950), who provided a conceptual framework for studying the trade effects of a FTA. Since Viner’s work, most of the other succeeding papers typically those by Cline (1978), Krueger (1995), Panagariya and Findlay (1994), Panagariya and Krishna (2002), Katsioloudes and Hadjidakis (2007) and Dominick (2007) also agreed that analysis of the static impact of a customs union can be fully extended to analyze static impact of a FTA. As pointed out by Viner (1950), the static impact is measured by trade creation and trade diversion and therefore, the welfare impacts a FTA is ambiguous, depending on whether trade diversion or trade creation overwhelms.

According to Viner (1950), Katsioloudes and Hadjidakis (2007), Nguyen (2011), Hoang et al. (2005), Plummer et al. (2010), Dominick (2007) and Negais (2009), trade creation occurs when domestic production in a FTA member is replaced by lower-cost production from another FTA member as a result of trade liberalization. In other words, there is a shift from the consumption of higher-price domestic products to lower-price products of other FTA members. The formation of a creation FTA therefore promotes trade between member states, improves the efficient allocation of resources and creates a greater specialization in producing comparative advantage goods. As a result, a creation FTA leads to the increase in consumer surplus and finally the welfare of member nations.

On the contrary, a FTA can divert trade flows due to its nature of discrimination between member nations and non-member nations. As tariff and non-tariff barriers are removed only within the FTA members, a FTA can make member nations divert imports from non-member nations into the member nations simply because the member countries enjoy preferential tariffs. On that ground, trade diversion occurs, worsening global resource allocation and shifting production away from comparative advantage. Therefore, a trade-diverting FTA leads to both trade creation and trade diversion, and can improve or worsen the welfare of members depending on the relative strength of these two opposing forces (Viner 1950; Dominick 2007; Katsioloudes and Hadjidakis 2007; Plummer et al. 2010; Nguyen 2011; Hoang et al. 2005; Negais 2009).

Besides the static effects, FTAs also bring about dynamic effects that take a longer time to be exposed in the economy but tend to continue generating benefits overtime even after the withdraw of a country from a FTA. The impact of FTAs on exploitation of economies of scale was confirmed by Evans et al. (2007), Katsioloudes and Hadjidakis (2007), Eicher et al. (2009) and Tran (2002). Furthermore, FTAs lead to other benefits such as promotion of specialization, competition, technology transfer, and improvement of efficiency as well as growth rate of the whole economy (Plummer et al. 2010; Eicher et al. 2009; Jha et al. 2010). With the development of new-generation FTAs, they also promote cooperation in other areas such as property right protection, job creation and sustainable development. Creating opportunities for member nations, especially developing countries, in reforming and harmonizing trade policies is another benefit that member nations seek for when joining a FTA (Katsioloudes and Hadjidakis 2007).

However, there are some challenges from FTAs that member nations should take into consideration. Firstly, from the social welfare perspective, a FTA is only the second best choice after multilateral liberalization due to its nature of discrimination against countries outside the FTA. Secondly, a FTA causes trade diversion and therefore can reduce welfare. Thirdly, participation in multiple FTAs at the same time leads to noodle bowl effects with complicated and overlapping rules of origin, and regulatory framework inconsistency, creating difficulties for governments in complying with FTAs and transaction costs for enterprises (Bui 2010; Multilateral Economic Cooperation Department 2009).

### Literature review on the impact of the EVFTA on Vietnam’s economy and trade

While the previous literature on the impact of FTAs, such as the ASEAN Trade in Goods Agreement (ATIGA), ASEAN-China FTA (ACFTA), Vietnam-Korea FTA (VKFTA), ASEAN-Japan comprehensive economic partnership agreement (AJEAP), ASEAN-Australia-New Zealand FTA (AANZFTA) and the TPP, on Vietnam’s economy in general and on trade in particular is intensive, there is a lack of papers focusing on trade impacts of the EVFTA. The EVFTA covers a big market with 28 partners, but most of the special attention by Vietnam’s enterprises and researchers has currently been prone to the TPP. One of the reasons for this situation is the lack of information and research on the impact of the EVFTA on Vietnam, especially sector trade impacts, while those on TPP are prevalent. Typical previous papers examining the impact of the EVFTA on Vietnam include Philip et al. (2011), Baker et al. (2014), Nguyen (2014a), Brauer et al. (2014) and Vu (2015). The first four papers focused on analyzing the effects of tariff reduction under the EVFTA on the whole Vietnam’s economy such as state budget, domestic demand, price, saving, investment, trade, employment and economic growth, and pointed out opportunities and challenges for Vietnam while Vu (2015) used trade indicators such as Revealed Comparative Advantage, Export Specialization and Trade Intensity to anticipate the potential benefits and losses of the EVFTA. Philip et al. (2011) and Baker et al. (2014) using the computable general equilibrium (CGE) also tried to estimate changes in Vietnam’s imports from EU in several sectors such as rice, garments, sugar, electronics, machinery, chemicals, transport and communication. Nguyen (2014a) adopted the gravity model to estimate changes in overall trade flows between two nations. Philip et al. (2011), Baker et al. (2014) and Nguyen (2014a) provided qualitative analysis of current development of some sectors such as automotive, electronics, garments, furniture, coffee and fisheries in the context of upcoming EVFTA. However, all of the above studies examined the impact of the EVFTA without taking into consideration of Vietnam’s integration into other FTAs and so far there is virtually no research investigating impacts of the EVFTA on Vietnam’s imports of pharmaceuticals.

Recent years have seen a great deal of papers concerning the development of Vietnam’s pharmaceutical industry. Nguyen (2014b), and Nguyen and Le (2015) analyzed the performance, market demands, products, enterprises and the competitive environment of the overall industry and examined the performance of some major players in the market. VCBS (2014) and EVBN (2014) focused its analysis on main features of Vietnam’s medicines market such as prices, types of medicines, distribution system, price management, materials and legal policies while Hoang (2014) tried to position Vietnam’s pharmaceutical industry in the world pharmaceutical map. All of the above authors agreed that Vietnam’s medicines industry is in the early state of development with small and low-competitive domestic firms, low investment, loose price management and intellectual property protection, and inadequate policies and mechanism for development of the sector. In addition, Vietnam’s pharmaceutical sector has been over-reliant on imported material inputs and pharmaceuticals (Nguyen 2014b), and overwhelmed by foreign companies in patent and specialty drug segments (EVBN 2014; VCBS 2014; Hoang 2014). Vietnam’s pharmaceutical enterprises have mainly produced generic drugs at low-value and with limited types of products (Maybank KimEng 2015; Nguyen 2014b; Vinapharm 2013). The authors, however, recognized the potentials for future development of the sector because of high demand, increasing health care expenditure (EVBN 2014; BMI 2016; HK 2014), higher access to the world medicine market (Vu 2014) and healthcare education improvement (Nguyen 2014b). One common feature of the above-mentioned papers is that although they were relatively successful at describing the current status and pointing out problems of Vietnam’s pharmaceutical sector, they ignored describing the aspects of imports and exports whereas Vietnam has largely reliant on imported medicines. Instead, imports and exports of Vietnam’s pharmaceuticals have unsystematically updated and analyzed just through short articles such as those written by Le (2015), Nguyen (2015), Quoc (2009), The (2015a) and The (2015b). The survey of last literature therefore shows that there is a lack of studies on Vietnam’s imports of pharmaceuticals at 6-digit level of Harmonized System (HS) and there is no study on the impact of the EVFTA on Vietnam’s pharmaceutical imports at disaggregated level.

In summary, review of the past literature reveals some important research gaps. Firstly, while the impact of FTAs including the TPP on Vietnam’s economy is prevalently investigated in the previous studies, the impact of the EVFTA has been ignored. Secondly, the previous studies related to the EVFTA focused its analysis on the impact on the whole economy rather than trade impacts. Thirdly, there is a lack of study estimating comprehensively trade impact of the EVFTA by sector at disaggregated level. Fourthly, the previous literature estimated impacts of the EVFTA in isolation with other FTAs of Vietnam. Finally but most importantly, there is so far virtually no study quantifying impacts of the EVFTA on Vietnam’s import of pharmaceuticals from the EU. This paper therefore contributes to the past literature by analyzing ex-ante trade impacts of tariff elimination commitments under the EVFTA on Vietnam’s import of pharmaceuticals from the EU at disaggregated level of HS 6 digits, taking into consideration of Vietnam’s integration in this sector with the ASEAN + 3 and TPP countries.

## An overview of Vietnam’s pharmaceutical imports from the EU

**Fig. 1:**
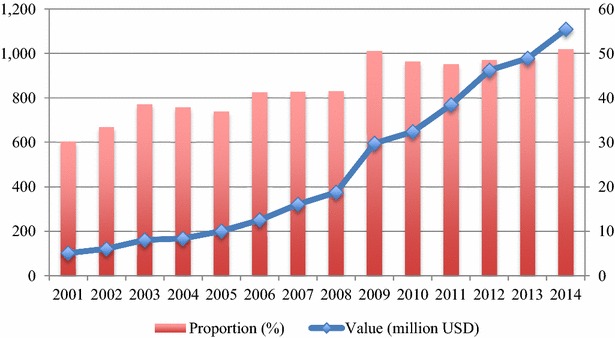
Value and proportion of Vietnam’s pharmaceutical imports from the EU, 2001–2014. *Source*: ITC (2016)

Pharmaceutical imports of Vietnam from the EU have a tendency to grow steadily during the period 2001–2014 despite of the global financial crisis as well as the economic instability of the EU in debt crisis. In 2014, Vietnams’ pharmaceutical imports reached USD 1,108 million, increasing by more than 10 times from USD 102 million in 2001 (Fig. 1). This upward trend originated mainly from the increasing healthcare expenditure of Vietnam and strengthened trade relationships between Vietnam and the EU, notably the signing of the EU—Vietnam Partnership and Cooperation Agreement in 2012 and the negotiations of the EVFTA from 2012.

In the period 2001–2014, the EU has consistently the biggest pharmaceutical import market of Vietnam with an increasing proportion (Fig. 1). In 2001, 30.15 % of Vietnam’s pharmaceutical imports came from the EU but in 2013 and 2014, this share increased to 48.82 and 50.98 % respectively. Besides the EU, other big partners Vietnam has imported medicines from include India, South Korea, Thailand and China with the proportions of around 12.4, 7.7, 3.2 and 2.8 % in 2014, respectively (ITC 2016). Pharmaceuticals are also the important imported commodity of Vietnam from the EU, ranking second after machinery and accounting for 12.5 % of Vietnam’s total imports from the EU in 2014.

**Fig. 2:**
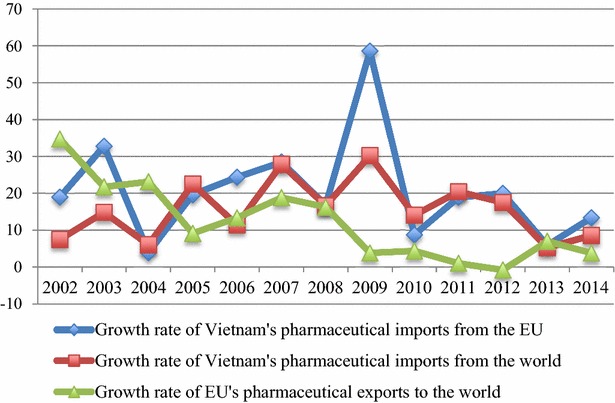
The growth rate of Vietnam and the EU’s pharmaceutical imports and exports, 2002-2014 (Unit: %). *Source*: Author’s calculations from ITC (2016)

In the past three years, Vietnam’s pharmaceutical imports from the EU also grew stronger than its imports from the rest of the world (Fig. 2), contributing to a strong increase in the EU’s market share in Vietnam. Besides, since 2005, the growth rates of the EU’s pharmaceutical exports to the world were always lower than that of Vietnam’s pharmaceutical imports from the EU, except in 2013. The increasing tendency in value and proportion, and high growth rate of Vietnam’s medicines imports from the EU show the growing dependence of Vietnam on the EU market. Therefore, when the EVFTA comes into effect, requiring Vietnam to remove tariffs for medicines imported from the EU, it will undeniably affect Vietnam’s medicines imports as well as the domestic market.

**Fig. 3:**
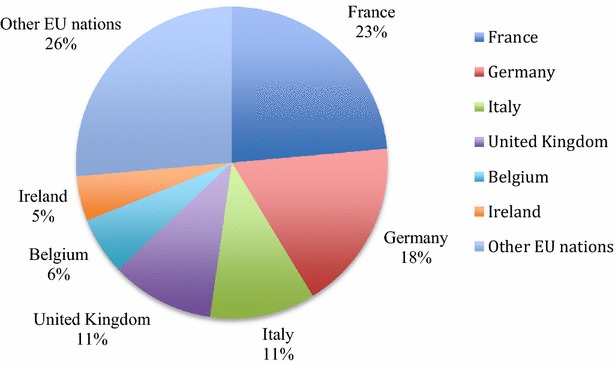
Pattern of Vietnam’s pharmaceutical imports by the EU partner in 2014 (Unit:  %). *Source*: Author’s calculations from ITC (2016)

From 2009 up to 2014, among the EU nations, France, Germany, Italy, the United Kingdom (UK), Belgium, and Ireland were Vietnam’s biggest pharmaceutical import markets (Additional file 1), which accounted for more than 73 % of Vietnam’s total pharmaceutical imports from the EU in 2014 (Fig. 3). Before 2009, besides these countries, Hungary and the Netherlands were also two major pharmaceutical sources for Vietnam. Vietnam virtually did not import from Estonia, Croatia, Luxembourg, Slovakia, and Latvia and started to import from Finland, Malta, Czech Republic and Lithuania several years ago. Therefore, there is a big disparity in Vietnam’s pharmaceutical imports by the EU nation, showing that the country heavily depends on some key EU’s markets.

Concerning the import pattern by group of product, Vietnam imported from the EU “medicaments mixtures in dosage” (HS 3004) the most. HS 3004 import value rose continuously over the years from nearly USD 94 million in 2001 to USD 954.17 million in 2014 (Additional file 2). Although the share of HS 3004 witnessed a slight decrease over the period 2001-2014, it was still at a high level to reach 86.10 % of total Vietnam’s pharmaceutical imports from the EU and 51.02 % of total Vietnam’s pharmaceutical imports from the world at the end of the period. In HS 3004 group, the biggest medicines imported from the EU were “medicaments nes” (HS 300490) such as analgesics, fever relief, anesthetics, anti-HIV, anti cancer, Parkinson, and cardiovascular medicines; followed by “antibiotics not containing penicillin, ampicillin, and amoxicillin” (HS 300420), “ antibiotics containing penicillin, ampicillin, and amoxicillin “ (HS 300410), and “hormones medicines” (HS 300439).

Ranking second is “human and animal blood, antisera, vaccines, toxins and micro-organism culture” (HS 3002), whose import values soared by 16 times from USD 5.61 million in 2001 to USD 89.88 million in 2014 (Additional file 2). Since 2009, Vietnam’s imports of HS 3002 has sharply increased to reach the share of 8.11 % of the total value of Vietnam’s pharmaceutical imports from the EU, accounting for more than 52 % of total value of Vietnam’s HS 3002 imports from the world in 2014. For this group, the biggest products imported from the EU were “vaccines for human use” (HS 300220) and “vaccines for veterinary use” (HS 300230).

Vietnam also imported from the EU a small amount of “medicament mixtures not in dosage” (HS 3003) and “pharmaceutical goods” (HS 3006) with the corresponding import turnovers of around USD 30.35 million and USD 31.48 million in 2014 (Additional file 2), accounting for only 2.74 and 2.84 % of total pharmaceutical imports of Vietnam from the EU. However, in comparison with overall imports of Vietnam from the world, more than 52.41 and 63.84 % of Vietnam’s HS 3003 and HS 3006 imports were from the EU. For HS 3006, Vietnam imported from the EU “opacifying preparation for x-ray and diagnostic reagents “(HS 300630) the most while “medicaments nes, formulated, in bulk” (HS 300390) was the biggest imported product in HS 3003.

Two remaining groups namely “dressing packaged for medical use” (HS 3005), and “glands and extracts, secretions for organotherapeutic uses, and heparin and its salts” (HS 3001) took small proportions of 0.20 and 0.01 % of Vietnam’s total drug imports from the EU, respectively. It is because most of the products in HS 3005 can be produced by the domestic pharmaceutical companies. In addition, production of HS 3005 drugs does not require high technology, thus, Vietnam can import them from non-EU countries at lower prices such as Thailand and China. Nearly 71.75 % of Vietnam’s HS 3005 imports were from these two countries while the share of imports from the EU accounted for only 9.38 % in 2014. For HS 3005 group, Vietnam imported from the EU “dressings and other articles having an adhesive layer” (HS 300510) the most.

Imports, preservation and utilization for therapy of HS 3001 products, especially glands and extracts, require high technology and costs. In the context of the low-tech pharmaceutical domestic sector and low supply of these products in the world, Vietnam has therefore imported little glands and extracts from the world, including the EU. Instead, for HS 3001, Vietnam imported most from EU “heparin and its salts” (HS 300190). In 2014, Vietnam’s imports of HS 3001 from the EU accounted for only 0.01 % of its total pharmaceutical imports from the EU and over 9.91 % of Vietnam’s HS 3001 imports from the world.

### Vietnam’s tariff reduction commitments on pharmaceutical imports from the EU

Vietnam imposed a relatively stable and low tariff on pharmaceuticals imported from the EU. From 2012 to 2014, the pharmaceutical tariff lines at 0 % nearly stayed the same, accounting for 62.63 and 63.64 % of total tariff lines respectively and the average tariff rate remained unchanged at 2.26 % despite slight changes in tariff rates of HS 3004 and HS 3005 (see Table 1 at the end of the text file). In general, Vietnam did not impose tariff on HS 3001 and HS 3002 while the rate on HS 3005 were at the highest level, increasing from 7 % in 2012 to 8 % in 2014.^1^ The remaining three groups namely HS 3003, HS 3004, and HS 3006 were protected with the tariff rates of 2.00, 2.22 and 2.67 % in 2012. In 2014, tariff rates imposed on HS 3003 and HS 3006 stayed the same but those on HS 3004 decreased minimally to 2.13 %.^2^

**Table 1:** Vietnam’s tariffs for pharmaceuticals imported from the EU *Source:* Author’s calculations from Vietnam’s tariff schedule in the EVFTA and Ministry of Finance (2013)

HS	Tariff lines (number)	Base year 2012			2014			Tariff reduction schedule under the EVFTA			
		Tariff lines at 0 % (number)	Tariff lines at 0 % (%)	Simple average tariff rate (%)	Tariff lines at 0 % (number)	Tariff lines at 0 % (%)	Simple average tariff rate	Tariff lines in Schedule A (%)	Tariff lines in Schedule B5 (%)	Tariff lines in Schedule B7 (%)	Tariff lines in Schedule B10 (%)
3001	2	2	2.02	0.00	2	2.02	0.00	2.02	0.00	0.00	0.00
3002	9	9	9.09	0.00	9	9.09	0.00	9.09	0.00	0.00	0.00
3003	8	6	6.06	2.00	6	6.06	2.00	6.06	0.00	2.02	0.00
3004	60	34	34.34	2.22	35	35.35	2.13	34.34	1.01	24.24	0.00
3005	5	0	0.00	7.00	0	0.00	8.00	0.00	0.00	5.05	0.00
3006	15	11	11.11	2.67	11	11.11	2.67	11.11	0.00	3.03	2.02
Total	99	62	62.63	2.26	63	63.64	2.26	62.63	1.01	34.34	2.02

On 1st February 2016, the full text of the EVFTA has been made public for information purposes and will be subject to legal revision for ratification. According to Vietnam’s tariff schedule^3^ disclosed so far, pharmaceutical tariff reductions are categorized into four groups: A, B5, B7 and B10 with the basic tariff rate of the negotiated year 2012. Accordingly, 62.63 % of pharmaceutical tariff lines are under Schedule A, where tariff rates shall be eliminated immediately on the date the EVFTA enters into force (Table 1). It is noted that Schedule A includes the tariff lines that were already at 0 % rate in the base year 2012. 1.01 % of tariff lines falls into Schedule B5, where tariff rates shall be removed in six equal annual stages beginning on the date the EVFTA comes into force. A large proportion of tariff lines, which is 33.34 %, are categorized into Schedule B7 to remove tariff in eight equal annual stages and the rest of 2.02 % into Schedule B10 with eleven equal-annual-stage of tariff removal starting on the date the EVFTA comes into effect.

More detailed, Vietnam’s tariff on the EU’s pharmaceuticals shall be eliminated in the 11th year from the date the EVFTA comes into force, cut from 2.26 % of the base year to 1.99 % in the first year, 0.61 % in the middle year and 0 % in the 11th year (Table 2). All of the products at 6-digit HS in HS 3001 and HS 3002, five of six in HS 3003, two of eight in HS 3004 and six of nine in HS 3006 were already at 0 % tariff rate, therefore the tariff reduction load on Vietnam would be left on HS 3004, HS 3005 and some products in HS 3006.

**Table 2:** Vietnam’s tariff reduction schedule on pharmaceuticals imported from the EU under the EVFTA at 6-digit HS (%) *Source:* Author’s calculations from Vietnam’s tariff schedule under the EVFTA

HS	Base year	1st year	2nd year	3rd year	4th year	5th year	6th year	7th year	8th year	9th year	10th year	11th year
**30**	**2.26**	**1.99**	**1.71**	**1.44**	**1.16**	**0.89**	**0.61**	**0.34**	**0.08**	**0.05**	**0.03**	**0.00**
***3001***	***0.00***	***0.00***	***0.00***	***0.00***	***0.00***	***0.00***	***0.00***	***0.00***	***0.00***	***0.00***	***0.00***	***0.00***
300110	0.00	0.00	0.00	0.00	0.00	0.00	0.00	0.00	0.00	0.00	0.00	0.00
300190	0.00	0.00	0.00	0.00	0.00	0.00	0.00	0.00	0.00	0.00	0.00	0.00
***3002***	***0.00***	***0.00***	***0.00***	***0.00***	***0.00***	***0.00***	***0.00***	***0.00***	***0.00***	***0.00***	***0.00***	***0.00***
300210	0.00	0.00	0.00	0.00	0.00	0.00	0.00	0.00	0.00	0.00	0.00	0.00
300220	0.00	0.00	0.00	0.00	0.00	0.00	0.00	0.00	0.00	0.00	0.00	0.00
300230	0.00	0.00	0.00	0.00	0.00	0.00	0.00	0.00	0.00	0.00	0.00	0.00
300290	0.00	0.00	0.00	0.00	0.00	0.00	0.00	0.00	0.00	0.00	0.00	0.00
***3003***	***2.00***	***1.75***	***1.50***	***1.25***	***1.00***	***0.75***	***0.50***	***0.25***	***0.00***	***0.00***	***0.00***	***0.00***
300310	5.33	4.67	4.00	3.33	2.67	2.00	1.33	0.67	0.00	0.00	0.00	0.00
300320	0.00	0.00	0.00	0.00	0.00	0.00	0.00	0.00	0.00	0.00	0.00	0.00
300331	0.00	0.00	0.00	0.00	0.00	0.00	0.00	0.00	0.00	0.00	0.00	0.00
300339	0.00	0.00	0.00	0.00	0.00	0.00	0.00	0.00	0.00	0.00	0.00	0.00
300340	0.00	0.00	0.00	0.00	0.00	0.00	0.00	0.00	0.00	0.00	0.00	0.00
300390	0.00	0.00	0.00	0.00	0.00	0.00	0.00	0.00	0.00	0.00	0.00	0.00
***3004***	***2.22***	***1.94***	***1.66***	***1.38***	***1.09***	***0.81***	***0.53***	***0.27***	***0.00***	***0.00***	***0.00***	***0.00***
300410	2.60	2.28	1.95	1.63	1.30	0.98	0.65	0.33	0.00	0.00	0.00	0.00
300420	2.50	2.19	1.88	1.56	1.25	0.94	0.63	0.31	0.00	0.00	0.00	0.00
300431	0.00	0.00	0.00	0.00	0.00	0.00	0.00	0.00	0.00	0.00	0.00	0.00
300432	1.67	1.46	1.25	1.04	0.83	0.63	0.42	0.21	0.00	0.00	0.00	0.00
300439	0.00	0.00	0.00	0.00	0.00	0.00	0.00	0.00	0.00	0.00	0.00	0.00
300440	1.88	1.64	1.41	1.17	0.94	0.70	0.47	0.23	0.00	0.00	0.00	0.00
300450	1.00	0.83	0.67	0.50	0.33	0.17	0.00	0.00	0.00	0.00	0.00	0.00
300490	2.59	2.26	1.94	1.62	1.29	0.97	0.65	0.32	0.00	0.00	0.00	0.00
***3005***	***7.00***	***6.13***	***5.25***	***4.38***	***3.50***	***2.63***	***1.75***	***0.88***	***0.00***	***0.00***	***0.00***	***0.00***
300510	7.00	6.13	5.25	4.38	3.50	2.63	1.75	0.88	0.00	0.00	0.00	0.00
300590	7.00	6.13	5.25	4.38	3.50	2.63	1.75	0.88	0.00	0.00	0.00	0.00
***3006***	***2.67***	***2.40***	***2.13***	***1.86***	***1.59***	***1.32***	***1.05***	***0.78***	***0.51***	***0.34***	***0.17***	***0.00***
300610	0.00	0.00	0.00	0.00	0.00	0.00	0.00	0.00	0.00	0.00	0.00	0.00
300620	0.00	0.00	0.00	0.00	0.00	0.00	0.00	0.00	0.00	0.00	0.00	0.00
300630	1.75	1.53	1.31	1.09	0.88	0.66	0.44	0.22	0.00	0.00	0.00	0.00
300640	0.00	0.00	0.00	0.00	0.00	0.00	0.00	0.00	0.00	0.00	0.00	0.00
300650	0.00	0.00	0.00	0.00	0.00	0.00	0.00	0.00	0.00	0.00	0.00	0.00
300660	0.00	0.00	0.00	0.00	0.00	0.00	0.00	0.00	0.00	0.00	0.00	0.00
300670	0.00	0.00	0.00	0.00	0.00	0.00	0.00	0.00	0.00	0.00	0.00	0.00
300691	5.00	4.38	3.75	3.13	2.50	1.88	1.25	0.63	0.00	0.00	0.00	0.00
300692	14.00	12.73	11.45	10.18	8.91	7.64	6.36	5.09	3.82	2.55	1.27	0.00

Tariff rates for the whole pharmaceutical sector at 2-digit HS are in bold

Tariff rates for six pharmaceutical groups at 4-digit HS are in bolditalics

This paper aims at assessing the effects of only tariff reduction under the EVFTA on Vietnam’s imports of pharmaceuticals because of the following reasons.

Firstly, at the global level, in most of the studies quantifying trade impacts of FTAs, assessment of tariff removal has been a necessity and considered the first-order effect before conducting any other assessment of non-tariff barriers, even though the impacts of non-tariff barriers might be higher. In fact, the committed tariff schedule is more transparent and predictable, therefore the impact results are easier to be quantified and more persuasive. The impacts of non-tariff barriers on the contrary are much more difficult to be predicted and quantified because non-tariff barriers are less stable, related to policies and regulations that could be adjusted by domestic laws, and not bounded as stringently as tariff barriers. Especially for pharmaceutical products which affect considerably human’s lives and health, the possibility of countries to adopt beyond the border policies and trade defence measures for the purpose of protecting the health of community is eligible in many cases. Therefore, even though impacts of tariff removal might be not so high, this assessment is treated as the first persuasive start to understand quantified changes in imports from FTAs.

Secondly, at the country level, together with trade liberalization trend, Vietnam’s tariff barriers are decreasing but concerns on these barriers in negotiating and implementing FTAs remain high. It is because Vietnam’s capacity to develop, adopt and monitor the complicated non-tariff barriers is limited, resulting to the fact that Vietnam has generally relied on tariff barriers to protect domestic industries. In addition, impacts of tariff are generally realized more quickly and clearly than impacts of non-tariff barriers. Therefore, the foremost concern of Vietnam’s enterprises whenever Vietnam joins a FTA is when and how much tariff would reduce, and how tariff reduction would affect imports (Nguyen 2014c). In responses to this context of Vietnam, assessment of tariff barriers should be the first consideration for understanding impacts of the EVFTA.

Thirdly, at the industry level, pharmaceuticals are the second biggest imported products of Vietnam from the EU. Furthermore, although the overall tariff Vietnam has imposed on the EU’s medicines are relatively low, the highest rates have been imposed on types of medicines Vietnam can produce domestically (HS 3005 and HS 3006) and on those Vietnam has imported most from the EU (HS 3004). Therefore, even though the total effects of tariff removal on Vietnam’s pharmaceutical imports might not be high, the distribution of the effects is more important. Understanding the impacts of tariff removal accordingly would help the government and enterprises to figure out the most vulnerable pharmaceutical products and then design appropriate strategies to prepare for the future EVFTA.

Fourthly, also at the industry level, because of the increasing role of pharmaceuticals in Vietnam-EU trade, there is a separate Annex for pharmaceutical products and medical devices in the EVFTA. According to this Annex, two parties commit the principles to conduct measures to facilitate bilateral pharmaceutical trade and the use of international standards, practices and guidelines as a basis for the technical regulations. Vietnam also agrees to allow the EU enterprises to participate into pharmaceutical bidding contracts and price negotiation, import pharmaceuticals and sell pharmaceuticals to distributors in Vietnam. These commitments would potentially remove substantially the long-lasing barriers for the EU enterprises, bringing about significant advantages for them over other foreign pharmaceutical enterprises. However, with all the information disclosed so far, it is still not clear about when, how and to what extent Vietnam will remove these barriers for the EU. Therefore, up to now, there is not enough information to assess impacts of the above mentioned non-tariff barriers on Vietnam’s pharmaceutical imports but it is more appropriate to focus at first the impacts of tariff removal. In addition, it is worth noting that patents on pharmaceuticals are considered more important than in most other sectors. However, in context of the EVFTA commitments, Vietnam and the EU have not agreed on pharmaceutical patents, and Vietnam has not successful at demanding the EU to reduce protection duration for patent medicines. Therefore, up to now, assessment of the effects of patents on pharmaceuticals in Vietnam might not be relevant. Last but not least, it is quite complicated to assess the impacts of the non-tariff barriers at disaggregated level since these barriers tend to affect across the different types of pharmaceutical products. It is unlike the tariff removal that clearly affects each type of medicines differently. Therefore, given the objectives of this paper to assess the impacts of the EVFTA at disaggregated level to understand the distribution effect of the EVFTA, it would be more appropriate to examine tariff removal as the first–order effect. The assessment of non-tariff barriers should be conducted when there are more information about how these barriers will be eliminated and how Vietnam’s pharmaceutical policy responses are to the EVFTA commitments.

## Methodology and data

### Methods

Staronova (2007) argued that impact assessment aims at providing scientific-based evidence for policy-making decision and can be conducted by two ways namely ex-ante impact assessment and ex-post impact assessment. The former is adopted to evaluate the potential impacts of policy changes that will be completed in a given point of time in the future while the later is used for policy changes that are already finished. As Vietnam and the EU have just concluded negotiations of the EVFTA that is expected to come into effort in 2018 at the soonest (Henriksson 2016; Ministry of Industry and Trade 2016; Delegation of the European Union to Vietnam 2016), ex-ante impact assessment is an appropriate choice for case of the EVFTA.

As stated by Kehoe and Kehoe (1994a), Mikic (2005), Plummer et al. (2010), Karingi et al. (2005a), and Philip et al. (2011), the ex-ante impact assessment of a FTA can be carried out by different methods but the most common ones include: (1) trade indicators; (2) the partial equilibrium through adoption of the SMART; and (3) the computable general equilibrium (CGE) through GTAP model (Global Trade Analysis Project). Each method can be used to evaluate specific aspects of impacts of a FTA and has its own advantages and disadvantages. Trade indicators are used to describe, evaluate and compare trade flows and pattern of a country overtime or across countries (Mikic 2005). As argued by Plummer et al. (2010) and Vu (2015), these indicators do not merely provide information on the current status of trade but are helpful in diagnosing potential impacts of a FTA. However, this method fails to provide the exact figures on impact of a FTA on trade and welfare, therefore, it is only regarded to be a first step to assess the future impact of a FTA. Among the methods adopted to assess impacts of trade policy changes, GTAP is so far the most comprehensive way in quantifying impacts of a FTA on different aspects of an economy such as GDP, trade, employment, investment, savings, price, and environment (Kehoe and Kehoe 1994b) because it stresses the interactions among sectors and markets (Nguyen 2014a). However, this method is quite complicated, requiring a wide range of data of all involved countries at both macroeconomic and industry level. In addition, as a CGE analysis, GTAP model also has its own disadvantages because it is constructed on ground of a series of complicated constraints and heavily depends on equilibrium conditions (Callaghan 2009; Cassing et al. 2010; Nguyen 2014a). More important, CGE cannot handle disaggregated data while a partial equilibrium model like the SMART allows evaluating impacts of a FTA at a much disaggregated product level (Admed 2010). The SMART also helps overcome the disadvantage of trade indicators approach in quantifying trade impacts of a FTA. One special advantage of the SMART model is that it allows quantifying impacts of tariff policy changes in a single market on trade flows, tariff revenue, trade creation effect, trade diversion effect, and social welfare of a nation detailed at HS 6-digit products (Cheong 2010; Admed 2010; Othieno and Shinyekwa 2011; Choudhry et al. 2013). Inference from results of the SMART simulation can also be good implications for both governments and enterprises in a given industry to prepare themselves for trade liberalization under a FTA. The appropriate selection of a research method should be based mainly on the objectives of the study. As this paper aims at estimating impacts of the EVFTA on Vietnam’s imports of pharmaceuticals at detailed level of 6-digit HS, the partial equilibrium through the SMART model therefore is the most suitable choice for this paper. However, it is noted that as a partial equilibrium model, the SMART also has it own limitations, by which the biggest one is to ignore economic interactions between different sectors in an economy. The model also neglects constraints on resources such as labor, land and capital, and movement of resources between sectors in an economy (Karingi et al. 2005a). Finally, the model does not return results on the effects on domestic production, which may be of interests to policy makers (Plummer et al. 2010).

Using the SMART model to analyze the future impact of a FTA is increasingly common due to the usefulness of this approach in assessing trade impacts at disaggregated level to provide better implications for governments and enterprises. For example, Othieno and Shinyekwa (2011) used this model to evaluate the future effects of the East African Community Customs Union on Uganda’s trade, tariff revenue and social welfare in sensitive products. With the objectives of assessing the likely economic and welfare impacts of the EU Partnership Agreement with the African countries at product level, Karingi et al. (2005a) and Karingi et al. (2005b) both applied the SMART model. Karingi et al. (2005a) used this model for the case of the EU-Africa Economic Partnership Agreement and Karingi et al. (2005b) for the EU-ECOWAS (Economic Community of West African States) Economic Partnership Agreement. Adopting the SMART model to stimulate the likely trade creation and diversion effects of the India-Sri Lanka FTA in three sectors including textiles, base metal and machinery equipment, Choudhry et al. (2013) saw a significantly higher trade creation than trade diversion and pointed out some products that seem to be benefited most for India from the agreement. Veeramani and Saini (2010) by using the SMART model found out that the ASEAN-India Preferential Trade Agreement led to a significant increase in India’s imports of coffee, tea and pepper from the ASEAN countries and the trade creation dominated over trade diversion.

In Vietnam, the number of studies adopting the SMART model for ex-ante impact assessment is still very limited. This partial equilibrium model was initiated to be used from Cassing et al. (2010), followed by Philip et al. (2011), Baker et al. (2014) and the most recent study of Tu and Le (2015). Cassing et al. (2010) used the SMART model to assess the future impacts of ASEAN + FTAs on Vietnam’s trade in some products such as footwear, fisheries, vegetable, electronics, cars, furniture and coffee while Tu and Le (2015) examined the likely impacts of Regional Comprehensive Economic Partnership Agreement on Vietnam’s trade at the disaggregated level of 6-digit HS. Aiming at assessing the potential impacts of the EVFTA on Vietnam’s trade, Philip et al. (2011) and Baker et al. (2014) made use of the SMART and CGE model. Philip et al. (2011) focused on Vietnam’s export of garments and footwear whereas Baker et al. (2014) extended the analysis to a wide range of products and found out that footwear and garments would receive the greatest gain from tariff liberalization under the EVFTA.

The objective of this paper is to assess ex-ante impact of the EVFTA on Vietnam’s pharmaceutical imports from the EU at 6-digit HS and decompose the total import changes into trade diversion and trade creation. From the past literature on advantages and disadvantages of different ex-ante impact assessment methods and the review of last studies adopting the SMART model, this model is proved to be an appropriate and useful methodology for this paper given its objectives.

### Data

The partial equilibrium SMART model and its simulation tool are part of the world integrated trade solution (WITS) database and software developed by the World Bank in conjunction with the United Nation Conference on Trade and Development. (UNCTAD) The SMART simulation requires data on trade values and tariffs faced by each exporting partner. In this paper, import values of Vietnam with the EU and the rest of the world were extracted from UN’s COMTRADE (common format for transient data exchange) and Trade Map database. The Most Favoured Nation (MFN) import tariff rates imposed by Vietnam on each partner were taken from UNCTAD’s TRAINS (trade analysis and information system), the WTO’s IDB (Integrated Data Base) and Ministry of Finance of Vietnam.

In addition, SMART requires three following parameters as inputs for the model: (1) import demand elasticity; (2) import substitution elasticity and (3) export supply elasticity. These elasticity come from the fact that the SMART model is developed based on the economic theories related to import demand and export supply with three important assumptions: (1) the Armington assumption of import demand side, (2) two-stage optimization process and (3) assumption of infinite export supply elasticity.

The import demand in the model is constructed based on the Armington assumption that commodities are differentiated by their origin countries. This assumption implies that there is an imperfect substitution between import sources and therefore, import demand does not completely shift to a FTA member although the FTA provides trade preferential. The SMART model also supposes that consumers make decisions on demand based on two-stage optimization process, which is related to the allocation of their expenditures by commodity and by import source (Amjadi et al. 2011; Admed 2010; Laird and Yeats 1986). At the first stage, consumers decide on how much to spend totally for imported goods on basis of import demand elasticity, whose values in the SMART model have been empirically estimated for each country and every HS 6-digt product based on Stern et al. (1976). At the next stage, they allocate expenditure among imported goods from different import sources based on their relative price. The change in allocation of expenditures by imported sources when the relative price changes is known as import substitution elasticity, which is supposed to be equal to 1.5 in the SMART model. For a given goods, different countries have to compete to export to an import market. The degree of responsiveness of the export supply to changes in the export price is given by the export supply elasticity. The SMART model assumes infinite export supply elasticity, implying that the export supply curves are flat and the world prices of each variety are exogenously given. Therefore, the export country can export as many as it can at a given world price. This is often called the price taker assumption, which is suitable for a small import country case. Under the assumption of infinite export supply elasticity, the SMART model allows estimating a quantity effect of a tariff reduction while the price effect is always equal to zero. By default, the SMART model uses 99 for an infinite elasticity for all products and partners (Amjadi et al. 2011; Veeramani and Saini 2010).

The Armington assumption of imperfect substitution is widely accepted in the previous studies applying the SMART model. This assumption is accordingly rational in the case of Vietnam and the EU because the pharmaceuticals of the EU are differentiated from those of the rest of the world and the EVFTA will not shift completely Vietnam’s pharmaceutical imports from the rest of the world to the EU. In addition, Vietnam is a small pharmaceutical importer in the world in general and with the EU in particular; therefore, the assumption of infinite elasticity embodied in the SMART model is also appropriate, showing that the increase in Vietnam’s pharmaceutical imports will not affect price in the EU. So, the value of 99 for export supply elasticity was adopted in this paper. The import substitution elasticity was set at 1.5 as specified in the SMART model because it is appropriate for industrial products as suggested by (Amjadi et al. 2011). The import demand elasticity detailed at 6-digit HS defaulted in the SMART were also applied in this paper because of its usefulness and accuracy at disaggregated level. In fact, using these elasticity parameters of the SMART model is a common approach used in the previous studies such as Cassing et al. (2010), Othieno and Shinyekwa (2011), Veeramani and Saini (2010), Karingi et al. (2005a, b), Philip et al. (2011), Baker et al. (2014), and Tu and Le (2015).

Finally, this paper adopted the HS classification, by which pharmaceuticals are divided into 6 groups of products namely: (1) HS 3001—glands and extracts, secretions for organotherapeutic uses, and heparin and its salts; (2) HS 3002—human and animal blood, antisera, vaccines, toxins and micro-organism culture; (3) HS 3003—medicament mixtures not in dosage; (4) HS 3004—medicaments mixtures in dosage; (5) HS 3005—dressing packaged for medical use and (6) HS 3006—pharmaceutical goods.

### Scenarios

Two scenarios were constructed based on Vietnam’s commitments of tariff reduction on pharmaceuticals under the EVFTA as well as the current pace of Vietnam’s integration in this sector with the rest of the world.

Vietnam and the EU signed the Declaration on the conclusion of the negotiations of the EVFTA in December 2015 and are conducting legal reviews to ratify this Agreement. It is expected that the EVFTA will enter into force in 2018. Based on this expectation and experience from the EU-Singapore FTA,^4^ the paper assumes that the EVFTA will come into force in 2018 and therefore Vietnam will complete to eliminate tariffs on pharmaceuticals from the EU by 2028. The base year for two scenarios is 2014.

- Scenario 1: Vietnam eliminates tariff on pharmaceuticals imported from the EU as scheduled from 2018 to 2028. Other integration process of Vietnam in the pharmaceutical sector with the rest of the world is not taken into consideration.

This scenario aims at examining the impact of the EVFTA on Vietnam’s pharmaceutical imports from the EU in isolation with integration of Vietnam in other FTAs to identify more clearly how the EVFTA will affect Vietnam.

- Scenario 2: Vietnam eliminates tariff on pharmaceuticals imported from the EU as scheduled from 2018 to 2028. In addition, Vietnam will also remove pharmaceuticals tariffs for TPP nations from 2018 to 2028 and for ASEAN + 3 (ASEAN and its three partners including China, South Korea and Japan) by 2028.

The TPP concluded recently requires Vietnam to open up the pharmaceutical market for the member countries. Up to now, like the EVFTA, the TPP is also expected to come into force in 2018 (WTO Center 2016; Nguyen 2016; Damodaran 2016). According to TPP’s full text that was already made public, the total duration for Vietnam to remove tariff for this sector would be 10 years, which is the same as that of the EVFTA.^5^ Therefore, the second scenario assumes that both the EVFTA and TPP will come into force in 2018 and Vietnam will finish removing tariffs for pharmaceuticals imported from the EU and TPP in 2028.

The commitments of Vietnam in the EVFTA and TPP in the pharmaceutical sectors are higher than that in other FTAs between Vietnam and ASEAN + 3 nations. While all tariff lines are eliminated under the EVFTA and TPP after ten years, Vietnam made the same commitment to reserve a peak tariff of 14 % for HS 300692 (waste pharmaceuticals) under ATIGA, ACFTA, VKFTA and the Vietnam-Japan Economic Partnership Agreement (VJEPA) (Ministry of Fanance 2014a, b; WTO Center 2013; Ministry of Industry and Trade 2014; WTO Center 2015). Vietnam also keeps positive tariff rates of 0–5 % for some tariff lines such as HS 30049099 in VJEPA and HS 30041016 in VKFTA. Therefore, this scenario optimistically assumes that under pressure of integration, ASEAN + 3 nations would try to keep up with the pace of liberalization in the TPP and EVFTA by removing pharmaceutical tariffs within the region.

Totally, scenario 2 assumed that the pharmaceutical tariffs Vietnam imposes on the EU, TPP and ASEAN + 3 are all 0 % in 2028. This scenario included 41 nations (Additional file 3), which provided more than 77.8 % of Vietnam’s imports of pharmaceuticals in 2014 (ITC 2016). Therefore, the scenario is expected to bring about a comprehensive and precise picture of the changes in Vietnam’s imports of pharmaceuticals from the EU when Vietnam integrates at the highest level in its existing key FTAs.

## Results

### Impacts of the EVFTA on overall changes in Vietnam’s pharmaceutical imports from the EU

The simulation results show that Vietnam’s pharmaceutical imports from the EU would increase in both scenarios (Table 3) because of two main reasons. Firstly, the EU’s pharmaceuticals would be cheaper than before when Vietnam removes tariffs under the EVFTA. Secondly, the EU’s pharmaceuticals would be less expensive than pharmaceuticals from the rest of the world or in other worlds the relative price of the EU’s pharmaceuticals would reduce.

**Table 3:** Overall changes in Vietnam’s pharmaceutical imports from the EU in two scenarios *Source:* Author’s calculations from SMART simulation results

Indicator	Scenario 1	Scenario 2
Initial import value (‘000 USD)	1,108,164	1,108,164
Import value in 2028 (‘000 USD)	1,142,273	1,138,937
Total import changes (‘000 USD)	34,109	30,773
Trade creation (‘000 USD)	17,639	17,639
Trade diversion (‘000 USD)	16,470	13,134
Increase in imports (%)	3.08	2.78
Trade creation/total import changes (%)	51.71	57.32

However, the tariff elimination would be likely not to affect considerably Vietnam’s imports of medicines from the EU because of relatively low level of initial tariff rates. In the first scenario when Vietnam only dismantles tariffs for the EU, Vietnam’s pharmaceutical imports from the EU would increase by 3.08 % compared to the initial level of the base year, equivalent to USD 34.11 million (Table 3). These figures for scenario 2 would be 2.78 % and USD 30.74 million respectively.

The increase in Vietnam’s medicines imports from the EU would be 9.8 % lower in scenario 2 than in scenario 1. It is because when Vietnam tries to integrate with not only the EU but also the TPP and ASEAN + 3 nations in scenario 2, Vietnam would shift a part of its pharmaceutical imports previously from the EU to TPP and ASEAN + 3. However, the reduction of 9.8 % implies that the deeper integration of Vietnam with two other groups would not result in a big decrease in Vietnam’s imports from the EU. Therefore, the EU would still be leading pharmaceutical market of Vietnam in the coming years.

### Impacts of the EVFTA by the EU country

There would be a significant difference in import value changes by partner in both scenarios. France would be the nation that Vietnam increases imports most, followed by Germany, the UK and Italy (Table 4). These four markets might account for around 69 % of additional imports of Vietnam from the EU in both scenarios. Belgium, Austria, Spain, Ireland and Sweden would together represent 19 % of Vietnam’s increased imports from the EU. The concentration of import increases in these countries could be mainly explained by their big initial trade with Vietnam. Another explanation might be the high production and export levels of pharmaceutical sectors witnessed in these countries for years (EFPIA 2014; ITC 2016). Therefore, there world be a great deal of opportunities for these countries to promote exports of medicines to Vietnam when the EVFTA comes into effect. On the contrary, Lithuania and Luxembourg could not increase their exports to Vietnam. Increases in Vietnam’s imports would be very low for other EU countries, which have been small pharmaceutical sources for Vietnam.^6^

**Table 4:** Changes in Vietnam’s pharmaceutical imports by the EU country *Source:* Author’s calculations from SMART simulation results

No.	Nation	Scenario 1			Scenario 2		
		Total import changes (‘000 USD)	Proportion in total import changes (%)	Growth (%)	Total import changes (‘000 USD)	Proportion in total import changes (%)	Growth (%)
1	France	8474	24.84	3.24	7677	24.95	2.94
2	Germany	6615	19.39	3.37	5993	19.48	3.05
3	UK	4276	12.54	3.59	3798	12.34	3.19
4	Italy	4187	12.28	3.46	3771	12.25	3.11
5	Belgium	1544	4.53	2.34	1387	4.51	2.10
6	Austria	1439	4.22	3.66	1308	4.25	3.33
7	Spain	1352	3.96	3.19	1220	3.97	2.88
8	Ireland	1096	3.21	2.13	994	3.23	1.93
9	Sweden	1030	3.02	3.62	939	3.05	3.30
10	Poland	797	2.34	2.75	710	2.31	2.45
11	Hungary	771	2.26	1.88	706	2.29	1.72
12	Cyprus	676	1.98	3.93	599	1.95	3.48
13	Netherlands	494	1.45	1.34	448	1.46	1.22
14	Bulgaria	396	1.16	3.53	355	1.15	3.16
15	Romania	247	0.72	3.78	224	0.73	3.43
16	Portugal	236	0.69	3.72	214	0.69	3.38
17	Greece	217	0.64	3.71	197	0.64	3.37
18	Slovenia	164	0.48	4.55	145	0.47	4.02
19	Denmark	39	0.12	0.17	36	0.12	0.16
20	Finland	18	0.05	2.55	17	0.05	2.35
21	Malta	16	0.05	3.66	14	0.05	3.33
22	Czech	13	0.04	1.46	12	0.04	2.71
23	Latvia	6	0.02	3.66	5	0.02	3.33
24	Slovak	5	0.01	3.66	4	0.01	3.33
25	Lithuania	0	0.00	0.09	0	0.00	0.00
26	Luxembourg	0	0.00	0.00	0	0.00	0.00
	Total	34,109	100.00	3.08	30,773	100	2.78

The growth rate of Vietnam’s pharmaceutical imports from France, Germany, the UK and Italy, which are also those who take up the biggest proportion of increases in Vietnam’s imports, would stay at the relatively high level of more than 3.2 % (Table 4). Some countries would have a potentially dynamic growth rate in exporting to Vietnam even though their initial trade values with Vietnam were not high such as Slovenia, Cyprus, Romania, Portugal and Greece. Pharmaceutical imports of Vietnam from Denmark, Czech Republic, Lithuania, Luxembourg and Netherland would growth at low level of below 1.5 % whereas from other countries of between 1.9 and 3.7 %.

### Impacts of the EVFTA by pharmaceutical group

The simulation results show that there would be an uneven distribution of Vietnam’s increased imports from the EU by pharmaceutical group. In both scenarios, nearly 98 % of increases in pharmaceutical imports of Vietnam from the EU would fall into HS 3004 (Table 5), reaching USD 33.2 million in scenario 1 and nearly USD 30 million in scenario 2. In comparison with scenario 1, Vietnam’s overall pharmaceutical imports from the EU decreases by USD 3.3 million in scenario 2, mainly because of the decrease in imports in this group of product. It implies that when Vietnam removes tariff for the EU, ASEAN + 3 and TPP, Vietnam would shift substantially its imports of HS 3004 to the TPP and ASEAN + 3 nations. These results could be explained by the fact that HS 3004 has been the key import product of Vietnam from both ASEAN + 3 and TPP. According to the SMART simulation results, Vietnam would import much more HS 3004 from the US and Australia in scenario 2 in comparison with scenario 1 (Additional file 4).

**Table 5:** Changes in Vietnam’s pharmaceutical imports from the EU by group of product *Source:* Author’s calculation from SMART simulation results

Group of product	Scenario 1			Scenario 2		
	Total import changes (‘000 USD)	Proportion in total import changes (%)	Growth (%)	Total import changes (‘000 USD)	Proportion in total import changes (%)	Growth (%)
HS 3001	0.00	0.00	0.00	0.00	0.00	0.00
HS 3002	0.00	0.00	0.00	0.00	0.00	0.00
HS 3003	36	0.11	0.12	36	0.12	0.12
HS 3004	33,262	97.52	3.49	29,995	97.47	3.14
HS 3005	380	1.11	17.01	365	1.19	16.35
HS 3006	431	1.26	1.37	377	1.22	1.20
Total	34,109	100.00	3.08	30,773	100.00	2.78

The increase in Vietnam’s imports of HS 3005 from the EU would be not high in value but grows at a rocket rate of around 17 % in both scenarios (Table 5). This high growth rate results from the initial high tariff rates Vietnam imposed on HS 3005. In scenario 2 when Vietnam removes tariff for all three groups of countries, its imports of HS 3005 from the EU would witness a slight decrease of about 3.9 % compared with that of scenario 1 and Vietnam would substitute a small part of HS 3005 imports from the EU for imports from the US, Korea, and Japan (Additional file 4). One notable feature is that Vietnam in this scenario would reduce substantially imports of HS 3005 from Thailand and China, which have been two biggest destinations of Vietnam for HS 3005. This can be explained by the fact that ASEAN and China already received 0 % tariff rate for this group of product from 2018, before the year that EVFTA and TPP are expected to come into effect. Therefore, when pharmaceuticals from the EU and TPP nations (excluding nations that are members of both ASEAN and TPP) are allowed to enter Vietnam at 0 % tariff rate, their prices relative to those of ASEAN and China nations would be lower than before, creating a price disadvantage for ASEAN and China in the Vietnamese market.

The increases in Vietnam’s HS 3006 imports from the EU would be modest in both value and growth rate in two scenarios (Table 5), equivalent to about USD 0.4 million at 1.2 % growth rate. However, Vietnam’s integration with the TPP and ASEAN + 3 nations would affect greater on Vietnam’s imports of HS 3006 than imports of HS 3005 from the EU. In fact, Vietnam’s imports of HS 3006 from the EU in scenario 2 decrease by 12.5 % compared to scenario 1 whereas this figure for HS 3005 is only 3.9 %. The US would be the new destination substituting substantially the EU in Vietnam’s imports of HS 3006. Japan and Canada are other replacements for the EU but at a lower level (Additional file 4).

The tariff removal for HS 3003 would result in a negligible increase in Vietnam’s imports of this group from the EU in both scenarios (Table 5). It is because HS 3003 has been a minor import commodity of Vietnam from the EU and been imposed the lowest tariff rate in comparison with other three tariff-imposed groups. In addition, there would be merely no change between scenario 1 and 2 in HS 3003 imports of Vietnam from the EU, implying that Vietnam’s integration with ASEAN + 3 and TPP would not affect its imports of HS 3003 from the EU. This comes from the fact that Vietnam has imported HS 3003 much lower from ASEAN + 3 and TPP nations than the EU, and almost all import tariff rates imposed on HS 3003 from ASEAN + 3 and TPP were already at 0 %.

In both scenarios, Vietnam would not change imports of HS 3001 and HS 3002 from the EU because all of the tariff lines for these two groups were already at 0 % in the base year.

### Impacts of the EVFTA by pharmaceutical product

The above analysis points out that there are three groups of pharmaceuticals that Vietnam should take into more careful consideration. They are HS 3004, which Vietnam would face with the highest increase in import values; HS 3006, which might be the second biggest changed groups; and HS 3005 whose imports could grow at a dynamic rate. Therefore, the paper analyzes more deeply the changes in imports of these groups at product level, on that ground identifying the most vulnerable products for Vietnam in the process of integrating with the EU in the pharmaceutical sector.

Among HS 4004, Vietnam would increase imports of HS 300490 at the largest such as antiseptics; anesthetics; painkiller; fever relief medicines; medicines containing acetylsalicylic, chlorpheniramine maleate, diclofenac and piroxicam; and especially specialty drugs (anti-cancer/HIV, diabetes and cardiovascular medicaments) that Vietnam so far could not produce. The increase in Vietnam’s imports of HS 300490 would take up of around 76 % of total additional imports from the EU in both scenarios (Table 6). When Vietnam eliminates tariffs for all three groups of countries in scenario 2, the EU would lose a part of HS 300490′s market in Vietnam to the US and Australia.

**Table 6:** Changes in Vietnam’s pharmaceutical imports from the EU by product *Source:* Author’s calculations from SMART simulation results

Product	Scenario 1			Scenario 2		
	Total import changes (‘000 USD)	Proportion in total import changes (%)	Growth (%)	Total import changes (‘000 USD)	Proportion in total import changes (%)	Growth (%)
HS 3004	33,262	97.52	3.49	29,995	97.47	3.14
300410	2275	6.67	5.25	1979	6.43	4.57
300420	4826	14.15	4.16	4129	13.42	3.56
300431	0	0.00	0.00	0	0.00	0.00
300432	468	1.37	2.08	454	1.47	2.02
300439	0	0.00	0.00	0	0.00	0.00
300440	45	0.13	4.32	45	0.15	4.32
300450	0	0.00	0.00	0	0.00	0.00
300490	25,647	75.19	3.65	23,388	76.00	3.33
HS 3005	380	1.11	17.01	365.0	1.19	16.35
300510	204	0.60	16.90	193	0.63	16.00
300590	176	0.52	17.15	172	0.56	16.77
HS 3006	431	1.26	1.37	377	1.22	1.20
300610	0.0	0.00	0.00	0	0.00	0.00
300620	0.0	0.00	0.00	0	0.00	0.00
300630	408	1.20	2.11	354	1.15	1.83
300640	0.0	0.00	0.00	0	0.00	0.00
300650	0.0	0.00	0.00	0	0.00	0.00
300660	0.0	0.00	0.00	0	0.00	0.00
300670	0.0	0.00	0.00	0	0.00	0.00
300691	23	0.07	6.70	23	0.07	6.64
300692	0.1	0.00	11.28	0	0.00	11.28
Total	34,109	100.00	3.08	30,773	100.00	2.78

Ranking second after HS 300490 in terms of the import increase would be HS 300420 (antibiotics not containing penicillin, ampicillin, and amoxicillin) and coming third would be HS 300410 (antibiotics containing penicillin, ampicillin, and amoxicillin). These two products together would account for about 20 % of total additional pharmaceutical imports of Vietnam from the EU in both scenarios (Table 6). In scenario 2, integration of Vietnam with the TPP and ASEAN + 3 nations would result in a relatively high reduction Vietnam’s imports of HS300410 and HS 300420 from the EU. Korea would be the key nation that replaces the EU’s pharmaceuticals in Vietnam for both HS 300410 and HS 300420, especially HS 300420. Thailand and China would be also other destinations for Vietnam to replace imports from the EU.

In both scenarios, HS 300432 (Adrenal cortex hormone and its derivatives) and HS 300630 (Opacifying preparation for x-ray and diagnostic reagents) would incur a small increase of more than 1 % in total additional imports of Vietnam from the EU (Table 6). In comparison with scenario 1, tariff elimination for all groups of countries in scenario 2 would result in a small decrease in Vietnam’s import from the EU for HS 300432 but a relatively big decrease of 13.2 % for HS 300630. Vietnam would shift its imports of HS 300432 from the EU mainly to Canada and HS 300630 to the US.

Besides, Vietnam’ import of HS 300510 (Dressings and other articles having an adhesive layer) and HS 300590 (Dressings and similar articles, impregnated or coated or packaged for medical use) would incur a significant growth rate of between 16 and 17 % compared to the initial levels (Table 6). These products have been highly protected by Vietnam for a long time. Therefore, the Vietnamese enterprise would face with higher competition from the EU for these two products when the EVFTA comes into force. Comparison of the changes in Vietnam’s imports from the EU in two scenarios points out that the effort of Vietnam’s in removing pharmaceutical tariffs for ASEAN + 3 and TPP would not affect considerably its imports of these two products from the EU.

### Trade creation and diversion effect

When Vietnam dismantles tariffs for the EU, its imports from the EU would increase in both scenarios as discussed above and the total additional imports could be decomposed into two parts namely trade creation and trade diversion.

Under the tariff reduction from the EVFTA, the EU pharmaceuticals would become cheaper than before and the EU pharmaceutical imports therefore would replace high-cost domestic production. The increase of Vietnam’s imports from the EU in this case is called trade creation that would be unchanged across scenarios. Trade creation improves welfare as domestic resources are allocated more efficiently but creates competition for the domestic producers.

In addition, the EVFTA would lower the price of the EU pharmaceuticals relative to pharmaceuticals from other part of the world. The increase in Vietnam’s imports from the EU due to reduction of the EU’s pharmaceutical relative price is called trade diversion, which lowers welfare because the low-cost production from the rest of the word is replaced by less efficient FTA member and production is forced to shift away from the comparative advantage.

The simulation results show that trade creation effect would be higher than trade diversion effect in both scenarios, implying that the EVFTA would improve welfares of Vietnam. When Vietnam removes tariff for only the EU, trade creation would account for 51.71 % of total trade effect (see Table 7 at the end of the text file). When the EU, ASEAN + 3 and TPP could export pharmaceuticals to Vietnam at 0 % tariff rate, the difference between price of the EU’s pharmaceuticals and pharmaceuticals from ASEAN + 3 and TPP would be narrowed and therefore lowering the trade diversion that Vietnam suffers from. The share of trade creation in total trade effect of scenario 2 would increase to 57.32 % from 51.71 % in scenario 1 and all of the affected EU nations would have trade creation effect that is higher than trade diversion effect. However, despite the improvement in trade creation ratio, trade creation still could not dominate trade diversion, showing that the improvement of Vietnam’s welfare would be not so high.

**Table 7:** Trade creation and trade diversion effect of the EVFTA *Source:* Author’s calculation from SMART simulation results

Nation	Trade creation		Scenario 1			Scenario 2		
	Trade creation (‘000 USD)	Share in total trade creation (%)	Total trade effect (‘000 USD)	Trade diversion (‘000 USD)	Share of trade creation in total trade effects (%)	Total trade effect (‘000 USD)	Trade diversion (‘000 USD)	Share of trade creation in total trade effects (%)
France	4506	25.55	8474	3968	53.18	7677	3171	58.70
Germany	3337	18.92	6615	3278	50.45	5993	2656	55.68
UK	2283	12.94	4276	1993	53.40	3798	1515	60.12
Italy	2138	12.12	4187	2049	51.06	3771	1633	56.70
Belgium	776	4.40	1544	768	50.27	1387	611	55.96
Austria	733	4.15	1439	706	50.91	1308	576	56.01
Spain	690	3.91	1352	662	51.02	1220	531	56.50
Ireland	573	3.25	1096	523	52.25	994	421	57.66
Sweden	516	2.93	1030	513	50.14	939	423	54.99
Hungary	395	2.24	771	376	51.28	710	316	55.45
Poland	394	2.23	797	404	49.36	706	311	56.00
Cyprus	343	1.94	676	334	50.66	599	256	57.21
Netherlands	249	1.41	494	245	50.37	448	199	55.57
Bulgaria	195	1.10	396	202	49.13	355	160	54.89
Romania	129	0.73	247	117	52.45	224	94	57.84
Portugal	117	0.66	236	118	49.73	214	96	54.85
Greece	111	0.63	217	106	51.14	197	86	56.27
Slovenia	104	0.59	164	60	63.55	145	40	72.08
Denmark	21	0.12	39	19	52.52	36	16	56.64
Finland	9	0.05	18	9	49.47	17	8	53.86
Malta	8	0.04	16	8	50.12	14	6	54.98
Czech Republic	7	0.04	13	6	52.63	12	5	58.05
Latvia	3	0.02	6	3	50.12	5	2	54.98
Slovak Republic	2	0.01	5	2	50.12	4	2	54.98
Lithuania	0	0.00	0	0	0.00	0	0	0.00
Luxembourg	0	0.00	0	0	0.00	0	0	0.00
Total	17,639	100.00	34,109	16,470	51.71	30,773	13,134	57.32

Trade creation effect would be unevenly distributed among the EU member countries, by which most of trade creation belongs to France, Germany, the UK and Italy in both scenarios (Table 7). In addition, besides France and the UK, Slovenia, Czech Republic, Denmark and Ireland would also receive the above-average share of trade creation in total trade effect. Another point of interest is to identify the non-EU countries whose trade is being replaced by imports from the EU as a result of the EVFTA. When Vietnam only removes tariff for the EU in scenario 1, India would be the biggest losers, followed by Korea, Switzerland and the US (Table 8). Vietnam’s imports of pharmaceuticals would also be diverted substantially from Thailand, China, Australia and Japan to the EU. In addition, among the top ten non-EU countries that would suffer from the largest extent of trade diversion, there would be seven nations that Vietnam at present have FTAs with including India, Korea, Thailand, China, Australia, Japan and Indonesia. It implies that the EVFTA potentially affects negatively on Vietnam’s integration with ASEAN + 3 in particular and the Asian region in general.

**Table 8:** Top ten countries suffering from trade diversion in scenario 1 (Unit: thousand USD) *Source:* SMART simulation results

No.	Nation	Trade diversion
1	India	−4581.05
2	Korea	−2479.76
3	Switzerland	−1704.77
4	United States	−1330.53
5	Thailand	−947.49
6	China	−862.12
7	Australia	−771.11
8	Japan	−487.13
9	Pakistan	−400.73
10	Indonesia	−375.99

## Discussion

From the above SMART simulation results, some following important implications are drawn to support Vietnam to well prepare for the upcoming EVFTA in the pharmaceutical sector.

Firstly, the SMART simulation results show that in overall tariff removal for the EU’s medicines would not result in a significant increase in Vietnam’s imports from the EU, but Vietnam’s deeper integration with ASEAN + 3 and TPP would only affect slightly on Vietnam’s imports from the EU. In scenario 1, Vietnam’s imports of pharmaceuticals from the EU would increase by 3.08 % while this figure for scenario 2 would be 2.78 %. Therefore, impacts of the EVFTA on Vietnam’s imports of pharmaceuticals would be relatively stable regardless of efforts of Vietnam in integrating with the rest of the world. In other words, it would be expected that in the near future, the EU would be still the most important and biggest source of pharmaceuticals for Vietnam. Therefore, both the Vietnamese government and pharmaceutical enterprises, especially the enterprises should pay more attention to the EVFTA and its impacts on the pharmaceutical sector instead of focusing too much on the TPP and neglecting the EVFTA as so far.

Secondly, in both scenarios, there might be an uneven distribution in Vietnam’s import increases among the EU nations. Around 60 % of increase in Vietnam’s pharmaceutical imports would be concentrated most in France, followed by Germany, the UK and Italy. Therefore, the paper argues that the biggest competition when the EVFTA comes into effect would come from France, Germany, the UK and Italy and this competition would be long-lasting because the increased imports of Vietnam from these countries are high in terms of both value and growth rate. For this reason, the Vietnamese enterprises should put a high priority on understanding the leading pharmaceutical enterprises from these countries in terms of their products, quality, development trend and especially strategy to penetrate the Vietnamese market. From the Vietnamese government side, the government should support the domestic enterprises in providing information on the pharmaceutical enterprises as well as the pharmaceutical industry of these four countries. These efforts are crucial for Vietnam’s enterprises to understand these biggest competitors to prepare themselves for at first competing successfully and then moving towards to mutual beneficial cooperation after the EVFTA enters into force.

Thirdly, the similar situation would occur with distribution of Vietnam’s additional imports by pharmaceutical product. The Vietnamese government and enterprises should perceive this uneven distribution in import increases at disaggregated level to design appropriate business and investment strategy. It is because this uneven distribution would lead to different level of competition among product. At a disaggregated level, Vietnam would increase imports of HS 300490 from the EU at the biggest. Other products with high level of the increased imports would be HS 300420, HS 300410, HS 300432 and HS 300630. Besides, Vietnam’s imports of HS 300510 and HS 300590 would grow quite rapidly at around 16 and 17 %. The above simulations results imply that the most vulnerable pharmaceutical product for Vietnam would be HS 300490, followed by HS 300420 and HS 300410. Therefore, for the domestic enterprises whose product portfolio focuses on these three products, it is of great importance to well prepare by improving their production capacity and investing more in R&D to move towards to higher quality products and specialty medicines. Taking advantage of the EVFTA to cooperate or create joint ventures with the EU companies in producing HS 300490, HS 300420 and HS 300410 is another option for the domestic enterprises. The Vietnamese government should make policies supporting for these domestic enterprises to improve their competitive capacity by developing pharmaceutical material areas and creating incentives for R&D activities in the pharmaceutical sector. For the domestic enterprises who focus on producing HS 300432, HS 300630, HS 300510 and HS 300590, the competition pressure they face with would be not as high as those who focus on HS 300490, HS 300410 and HS 300420. However, they should keep improving quality and variety of products and at the same time reducing the price to serve the domestic demand, especially for HS 300510 and HS 300910 that have been highly protected by the government for a long time. Otherwise, the threat to lose market to the EU pharmaceutical producers would possibly come true.

Fourthly, trade creation effect would be higher than trade diversion effect, representing 51.71 % of total trade effect in scenario 1. It implies that the EVFTA would improve welfare of Vietnam although the welfare improvement might be not too high. The welfare would potentially increase more when Vietnam removes tariffs for also the TPP and ASEAN + 3 nations because trade creation share increases to 57.32 % in scenario 2. In addition, the SMART simulation results show that if Vietnam only removes tariff for the EU, the EVFTA would negatively affect Vietnam’s integration in ASEAN and ASEAN + 3. Seven nations that Vietnam at present have FTAs with including India, Korea, Thailand, China, Australia, Japan and Indonesia are among the top ten non-EU countries that would suffer from the largest extent of trade diversion. This impact would deteriorate Vietnam’s ongoing integration effort in ASEAN. Therefore, Vietnam should promote the integration in the pharmaceutical sector with all three groups of nations, especially ASEAN and ASEAN’s key partners, to reduce trade diversion effect and raise the welfare of Vietnam by offering 0 % tariff rate for all of these countries, given that Vietnam should consider carefully the point of time to remove tariff for each group to avoid the sudden increase in its pharmaceutical imports.

Fifthly, if Vietnam removes pharmaceutical tariffs for all three groups of nations, besides the biggest competition from the EU, Vietnam should prepare for competition from other partners, especially the US and Australia. For HS 3004, the US and Australia would be other two big competitors; for HS 3005 would be South Korea and the US; and for HS 3006 the US. At the more disaggregated level, both TPP and ASEAN + 3 would affect considerably Vietnam’s import of HS 300490 from the EU, therefore, together with the EU, the US and Australia would be the biggest competitors. South Korea, followed by Thailand and China, would be the key destinations that would replace the EU’s pharmaceutical imports in Vietnam for HS 300410 and HS 300420. For HS 300432 and HS 300630, the competition from Canada and the US respectively would be intensive.

Finally, the SMART results also imply that Vietnam would continuously rely on imports of pharmaceuticals from the key EU partners after the EVFTA like France, Germany, Italy and the UK as the increased imports of Vietnam from these countries are high in both value and growth rate. In the current context when the EU has been trying to overcome a wide range of economic and political difficulties, and the UK is standing in front of leaving or staying the EU, there would be possibility that the exports of the EU’s pharmaceutical producers, led by the German, French and UK enterprises, to the world could be instable. Accordingly, Vietnam will be forced to divert its imports to other pharmaceutical markets such as Australia, South Korea, China, and Thailand. This diversion may put the Vietnamese pharmaceutical market into vulnerability and instability, affecting negatively on Vietnam’s economy due to the vital role of this product to health and life of the people. Therefore, Vietnam on the one hand perceives the competition from the EU’s pharmaceuticals when the EVFTA comes into force, on the other hand should realize that this agreement would be an opportunity for Vietnam to increases the access to high-quality medicines, especially patent medicines, from the EU at a lower price in the possibility of declining exports from the EU in difficult period of time. On that ground, Vietnam should take measures to diversify its import markets and consider importing medicines from other European markets such as Slovenia, Czech Republic, Denmark and Ireland, whose trade creation take a high proportion in total trade affects as specified in SMART simulation results. These four countries have also been large pharmaceutical exporters in the world. This market diversification would enable Vietnam to less depend on the traditional key EU partners and improve welfare while taking opportunity from the EVFTA. It requires the Vietnamese enterprises to learn more about these markets to identify the types of specific medicines that must be put priority to be imported given the strength of each market and Vietnam’s demand.

This paper has contributed to the existing literature by using the SMART model to analyze the impacts of the EVFTA on Vietnam’s imports of pharmaceuticals from the EU and propose some recommendations at a disaggregated level. However, it still has limitation and can be improved in the future. In fact, due to the non-existence of the empirical work to estimate the import demand elasticity, the import substitution elasticity and export supply elasticity for Vietnam, the paper used the elasticity values of the SMART model. Even though this approach has been commonly used by the previous studies, the future research would produce better results if using the elasticity values of the country whose pharmaceutical sectors are similar to Vietnam’s instead of adopting the SMART value. Moreover, this paper assessed impacts of tariff removal under the EVFTA. However, the non-tariff barriers are also obstacles to the EU when exporting and investing in Vietnam in the pharmaceutical sector. Therefore, when the commitments between Vietnam and the EU for this sector are clearly made public, the future research should take into consideration of the impacts of both tariff and non-tariff barriers to provide more precise estimations on Vietnam’s changes in pharmaceutical imports of the EU.

## Conclusions

By using the SMART model, this paper assessed ex-ante impacts of tariff removal under the EVFTA on Vietnam’s imports of pharmaceuticals from the EU based on two scenarios, which were constructed based on Vietnam’s tariff schedule disclosed so far under the EVFTA and the broader picture of on-going integration of the country in the pharmaceutical sector. In scenario 1, Vietnam would remove tariff for only pharmaceuticals imported from the EU while scenario 2 extends the coverage of tariff reduction to also ASEAN + 3 and TPP nations.

The results show that the EVFTA would result in an increase of about 3 % in Vietnam’s pharmaceutical imports from the EU and the EU would still be the biggest source of pharmaceuticals for Vietnam despites the efforts of Vietnam to integrate with the ASEAN + 3 and TPP nations in this sector. The uneven distribution in Vietnam’s pharmaceutical imports from the EU by nation, pharmaceutical group and pharmaceutical product would occur when the EVFTA is implemented. Most of the import increase would concentrate on France, Germany, the UK and Italy in terms of import source; on HS 3004 in term of pharmaceutical group; and on HS 300490, 300420, HS 300410, HS 300432 and HS 300630 in terms of pharmaceutical product. In addition, the EVFTA would potentially increase welfare of Vietnam because trade creation is bigger than trade diversion. However, the EVFTA would affect negatively Vietnam’s integration into ASEAN + 3 if Vietnam only removes pharmaceutical tariff for the EU nations. The above findings are of great importance because it provides strong evidence for Vietnam to pay more attention to the EVFTA and its impacts on pharmaceutical imports. The paper also suggests evidence-based implications for both Vietnam’s government and enterprises to overcome the possible challenges as well as make the potential opportunities from the EVFTA come true.

## Additional files

10.1186/s40064-016-3200-7 Pharmaceutical imports of Vietnam by EU’s partner (Unit: million USD).
10.1186/s40064-016-3200-7 Vietnam’s pharmaceutical imports from the EU by group of product, 2001-2014 (Unit: Million USD).
10.1186/s40064-016-3200-7 List of countries in scenario 2.
10.1186/s40064-016-3200-7 Changes in Vietnam’s pharmaceutical imports from the EU, ASEAN + 3 and TPP nations by pharmaceutical group (thousand USD).

## Abbreviations

AANZFTA: ASEAN-Australia-New Zealand free trade agreement
ACFTA: ASEAN-China free trade agreement
AJEPA: ASEAN-Japan comprehensive economic partnership agreement
ASEAN: Association of South East Asian Nations
ASEAN + 3: Association of South East Asian Nations and its three partners including China, South Korea and Japan
CGE: Computable general equilibrium
COMTRADE: Common format for transient data exchange
EVFTA: The European Union and Vietnam free trade agreement
FTA: Free trade agreement
GTAP: Global trade analysis project
HS: Harmonized system
HS 3001: Glands and extracts, secretions for organotherapeutic uses, and heparin and its salts
HS 3002: Human and animal blood, antisera, vaccines, toxins and micro-organism culture
HS 3003: Medicament mixtures not in dosage
HS 3004: Medicaments mixtures in dosage
HS 3005: Dressing packaged for medical use
HS 3006: Pharmaceutical goods
IDB: Integrated data base
MFN: Most Favoured Nation
R&D: Research and development
SMART: The software on market analysis and restrictions on trade
TPP: Trans-Pacific Partnership
TRAINS: Trade analysis and information system
UK: The United Kingdom
UNCTAD: United Nation conference on trade and development
US: The United States
VJEPA: Vietnam-Japan economic partnership agreement
VKFTA: Vietnam-Korea free trade agreement
WITS: World integrated trade solution
WTO: World trade organization

## Footnotes

1 All of the five tariff lines in HS 3005 increased from 7 to 8 % from 2012 to 2014.

2 Reduction in the tariff rate imposed on HS 3004 was due to a decrease from 5 to 0 % imposed on HS 30045021. All of other tariff lines in HS 3004 remained unchanged.

3 A detailed tariff schedule of Vietnam in the EVFTA can be seen at http://portal.moit.gov.vn/fta/App_File/FTA/en/02.%20Schedules%20of%20commitments/03%20-%20Tariff%20schedule%20of%20Viet%20Nam/2cii%20-%20Tariff%20Schedule%20of%20Viet%20Nam.pdf.

4 The EU and Singapore launched negotiation of the EU-Singapore FTA in March 2010. The Goods and Services Agreement was concluded in 2012 and the Investment Chapter in 2014. However, it is expected that two parties will ratify the Agreement after 2016. Thus, it takes around 3 years for an FTA with the EU to come into effort from the date of conclusion of the negotiation.

5 Tariff schedule of Vietnam under the TPP can be seen at https://ustr.gov/sites/default/files/TPP-Final-Text-Viet-Nam-Tariff-Elimination-Schedule.pdf.

6 Two countries namely Croatia and Estonia were not included in the model because of insufficient data. Therefore, estimations from SMART simulations might be lower than the real changes. However, this difference is expected to be marginal as Croatia and Estonia were small pharmaceutical import markets of Vietnam.
