# Supplementary material for: Assessing potential impacts of the EVFTA on Vietnam’s pharmaceutical imports from the EU: an application of SMART analysis
Source: Springerplus. 2016 Sep 7;5(1):1503. doi: 10.1186/s40064-016-3200-7 (PMC5014775; doi:10.1186/s40064-016-3200-7)
Supplement: Supplementary file 1 — 10.1186/s40064-016-3200-7 Pharmaceutical imports of Vietnam by EU’s partner (Unit: million USD). [file 40064_2016_3200_MOESM1_ESM.docx]

**Additional file 1 Pharmaceutical imports of Vietnam by EU's partner (Unit: million USD)**

| **Exporter** | **2001** | **2002** | **2003** | **2004** | **2005** | **2006** | **2007** | **2008** | **2009** | **2010** | **2011** | **2012** | **2013** | **2014** |
| --- | --- | --- | --- | --- | --- | --- | --- | --- | --- | --- | --- | --- | --- | --- |
| World | 339.56 | 364.92 | 419.07 | 444.40 | 544.56 | 606.65 | 775.77 | 905.01 | 1178.48 | 1344.04 | 1618.82 | 1902.46 | 2002.25 | 2173.63 |
| EU 28 | 102.38 | 121.82 | 161.75 | 168.16 | 201.21 | 250.21 | 321.46 | 375.44 | 595.27 | 647.30 | 769.67 | 923.53 | 977.51 | 1108.17 |
| France | 57.54 | 63.84 | 73.42 | 76.20 | 86.92 | 114.10 | 141.23 | 153.89 | 209.25 | 216.20 | 253.79 | 270.31 | 266.61 | 261.22 |
| Germany | 9.89 | 13.18 | 16.46 | 18.12 | 20.43 | 25.10 | 39.77 | 44.24 | 92.05 | 100.81 | 117.63 | 147.49 | 153.22 | 196.55 |
| Italy | 2.36 | 3.40 | 5.83 | 7.55 | 9.69 | 11.56 | 15.23 | 16.61 | 41.82 | 58.51 | 69.02 | 95.51 | 98.51 | 121.14 |
| UK | 0.85 | 7.30 | 15.82 | 15.81 | 15.66 | 18.17 | 18.65 | 27.44 | 51.88 | 49.58 | 60.33 | 77.94 | 81.61 | 119.18 |
| Belgium | 0.24 | 1.83 | 6.53 | 6.07 | 8.68 | 11.82 | 12.75 | 12.72 | 37.43 | 45.65 | 50.97 | 67.90 | 76.55 | 66.07 |
| Ireland | 0.01 | 1.30 | 0.51 | 0.32 | 1.18 | 2.23 | 7.09 | 14.69 | 26.45 | 29.42 | 31.02 | 45.67 | 60.65 | 51.38 |
| Spain | 0.48 | 0.93 | 1.70 | 2.50 | 3.66 | 4.98 | 6.52 | 8.35 | 17.08 | 18.81 | 23.21 | 32.31 | 43.69 | 42.37 |
| Hungary | 11.62 | 9.99 | 13.93 | 12.40 | 13.82 | 16.01 | 17.85 | 21.82 | 24.89 | 22.22 | 32.04 | 32.44 | 33.10 | 40.99 |
| Austria | 9.73 | 6.56 | 5.53 | 7.21 | 8.87 | 8.98 | 12.67 | 12.44 | 20.22 | 22.87 | 27.65 | 26.69 | 32.26 | 39.34 |
| Netherlands | 4.49 | 5.85 | 9.67 | 8.49 | 12.80 | 14.87 | 18.93 | 25.05 | 17.85 | 21.57 | 27.05 | 34.55 | 30.01 | 36.77 |
| Poland | 2.12 | 2.55 | 2.98 | 2.95 | 4.17 | 5.00 | 7.01 | 10.08 | 13.77 | 12.47 | 14.56 | 16.25 | 15.71 | 28.97 |
| Sweden | 0.06 | 0.68 | 3.07 | 4.21 | 5.61 | 6.79 | 8.84 | 11.43 | 16.71 | 23.66 | 27.58 | 33.19 | 33.13 | 28.46 |
| Denmark | 0.19 | 0.65 | 0.99 | 0.73 | 0.50 | 1.69 | 4.20 | 3.76 | 5.68 | 6.52 | 11.91 | 16.65 | 20.53 | 22.86 |
| Cyprus | 1.97 | 3.09 | 3.62 | 3.92 | 6.19 | 6.73 | 7.83 | 9.05 | 11.94 | 10.30 | 10.37 | 10.74 | 9.62 | 17.19 |
| Bulgaria | 0.24 | 0.14 | 0.45 | 0.51 | 0.34 | 0.38 | 0.63 | 0.52 | 1.00 | 2.26 | 2.63 | 3.49 | 5.67 | 11.23 |
| Romania | 0.00 | 0.00 | 0.00 | 0.01 | 0.12 | 0.21 | 0.69 | 1.39 | 0.83 | 0.57 | 2.35 | 5.01 | 6.78 | 6.52 |
| Portugal | 0.00 | 0.01 | 0.01 | 0.04 | 0.00 | 0.01 | 0.04 | 0.11 | 0.08 | 0.27 | 0.47 | 1.06 | 3.78 | 6.33 |
| Greece | 0.04 | 0.09 | 0.16 | 0.05 | 0.49 | 0.07 | 0.03 | 0.20 | 0.93 | 1.01 | 1.37 | 1.72 | 1.56 | 5.86 |
| Slovenia | 0.01 | 0.07 | 0.43 | 0.28 | 0.47 | 0.70 | 0.88 | 1.04 | 2.42 | 2.44 | 3.42 | 2.21 | 2.62 | 3.61 |
| Finland | 0.00 | 0.01 | 0.00 | 0.00 | 0.00 | 0.03 | 0.01 | 0.03 | 0.62 | 0.33 | 0.43 | 0.57 | 0.53 | 0.71 |
| Malta | 0.00 | 0.00 | 0.00 | 0.00 | 0.00 | 0.00 | 0.00 | 0.00 | 0.00 | 0.00 | 0.00 | 0.03 | 0.30 | 0.43 |
| Czech | 0.15 | 0.21 | 0.30 | 0.72 | 1.56 | 0.74 | 0.58 | 0.36 | 0.77 | 0.96 | 1.65 | 1.19 | 0.87 | 0.43 |
| Lithuania | 0.00 | 0.00 | 0.00 | 0.00 | 0.00 | 0.00 | 0.00 | 0.20 | 1.39 | 0.85 | 0.01 | 0.01 | 0.06 | 0.27 |
| Latvia | 0.00 | 0.00 | 0.00 | 0.00 | 0.00 | 0.00 | 0.00 | 0.00 | 0.00 | 0.00 | 0.00 | 0.00 | 0.00 | 0.16 |
| Slovakia | 0.39 | 0.13 | 0.33 | 0.06 | 0.00 | 0.00 | 0.00 | 0.00 | 0.20 | 0.00 | 0.23 | 0.36 | 0.03 | 0.13 |
| Luxembourg | 0.00 | 0.00 | 0.00 | 0.00 | 0.00 | 0.00 | 0.00 | 0.00 | 0.00 | 0.00 | 0.00 | 0.00 | 0.00 | 0.01 |
| Croatia | 0.00 | 0.00 | 0.02 | 0.01 | 0.05 | 0.05 | 0.03 | 0.02 | 0.01 | 0.00 | 0.00 | 0.24 | 0.12 | 0.00 |
| Estonia | 0.00 | 0.00 | 0.00 | 0.00 | 0.00 | 0.00 | 0.00 | 0.00 | 0.00 | 0.04 | 0.00 | 0.00 | 0.00 | 0.00 |

*Source*: ITC (2016)
