# Supplementary material for: Assessing potential impacts of the EVFTA on Vietnam’s pharmaceutical imports from the EU: an application of SMART analysis
Source: Springerplus. 2016 Sep 7;5(1):1503. doi: 10.1186/s40064-016-3200-7 (PMC5014775; doi:10.1186/s40064-016-3200-7)
Supplement: Supplementary file 2 — 10.1186/s40064-016-3200-7 Vietnam’s pharmaceutical imports from the EU by group of product, 2001-2014 (Unit: Million USD). [file 40064_2016_3200_MOESM2_ESM.docx]

**Additional file 2 Vietnam's pharmaceutical imports from the EU by group of product, 2001-2014 (Unit: Million USD)**

| **HS** | **2001** | **2002** | **2003** | **2004** | **2005** | **2006** | **2007** | **2008** | **2009** | **2010** | **2011** | **2012** | **2013** | **2014** |
| --- | --- | --- | --- | --- | --- | --- | --- | --- | --- | --- | --- | --- | --- | --- |
| 3001 | 0.14 | 0.01 | 0.04 | 0.10 | 0.00 | 0.15 | 0.15 | 0.02 | 0.01 | 0.01 | 0.12 | 0.03 | 0.08 | 0.06 |
| 3002 | 5.61 | 7.75 | 10.44 | 10.46 | 14.15 | 17.72 | 18.71 | 13.25 | 49.41 | 61.77 | 71.92 | 86.27 | 87.75 | 89.88 |
| 3003 | 0.93 | 2.25 | 2.61 | 3.23 | 4.20 | 4.16 | 7.44 | 10.85 | 15.13 | 16.83 | 21.47 | 24.30 | 31.08 | 30.35 |
| 3004 | 93.99 | 109.03 | 144.88 | 152.36 | 180.21 | 225.47 | 290.85 | 344.74 | 514.66 | 552.19 | 655.43 | 786.80 | 832.59 | 954.17 |
| 3005 | 0.72 | 0.97 | 0.53 | 0.06 | 0.02 | 0.10 | 0.05 | 0.12 | 0.72 | 0.81 | 0.61 | 0.92 | 1.79 | 2.23 |
| 3006 | 0.99 | 1.81 | 3.26 | 1.95 | 2.62 | 2.61 | 4.26 | 6.45 | 15.34 | 15.69 | 20.13 | 25.21 | 24.23 | 31.48 |
| **Total** | **102.39** | **121.83** | **161.76** | **168.16** | **201.20** | **250.21** | **321.46** | **375.43** | **595.27** | **647.30** | **769.67** | **923.54** | **977.51** | **1108.16** |

*Source:* ITC (2016)
