# Supplementary material for: Assessing potential impacts of the EVFTA on Vietnam’s pharmaceutical imports from the EU: an application of SMART analysis
Source: Springerplus. 2016 Sep 7;5(1):1503. doi: 10.1186/s40064-016-3200-7 (PMC5014775; doi:10.1186/s40064-016-3200-7)
Supplement: Supplementary file 3 — 10.1186/s40064-016-3200-7 List of countries in scenario 2. [file 40064_2016_3200_MOESM3_ESM.docx]

**Additional file 3 List of countries in scenario 2**

| **No.** | **Nation** | **Group** |
| --- | --- | --- |
| 1 | Austria | EU |
| 2 | Belgium |  |
| 3 | Bulgaria |  |
| 4 | Cyprus |  |
| 5 | Czech Republic |  |
| 6 | Denmark |  |
| 7 | Finland |  |
| 8 | France |  |
| 9 | Germany |  |
| 10 | Greece |  |
| 11 | Hungary |  |
| 12 | Ireland |  |
| 13 | Italy |  |
| 14 | Latvia |  |
| 15 | Lithuania |  |
| 16 | Luxembourg |  |
| 17 | Malta |  |
| 18 | Netherlands |  |
| 19 | Poland |  |
| 20 | Portugal |  |
| 21 | Romania |  |
| 22 | Slovak Republic |  |
| 23 | Slovenia |  |
| 24 | Spain |  |
| 25 | Sweden |  |
| 26 | United Kingdom |  |
| 27 | Australia | ASEAN+3 and TPP |
| 28 | Canada |  |
| 29 | Chile |  |
| 30 | China |  |
| 31 | Indonesia |  |
| 32 | Japan |  |
| 33 | Korea |  |
| 34 | Malaysia |  |
| 35 | Mexico |  |
| 36 | New Zealand |  |
| 37 | Peru |  |
| 38 | Philippines |  |
| 39 | Singapore |  |
| 40 | Thailand |  |
| 41 | United States |  |

Note: In the EU, two countries namely Croatia and Estonia were not included in the model because of insufficient data. In ASEAN+3, Brunei, Cambodia, Myanmar, and Laos were not included in the model because Vietnam has not imported pharmaceuticals from these countries.
