# Supplementary material for: Assessing potential impacts of the EVFTA on Vietnam’s pharmaceutical imports from the EU: an application of SMART analysis
Source: Springerplus. 2016 Sep 7;5(1):1503. doi: 10.1186/s40064-016-3200-7 (PMC5014775; doi:10.1186/s40064-016-3200-7)
Supplement: Supplementary file 4 — 10.1186/s40064-016-3200-7 Changes in Vietnam’s pharmaceutical imports from the EU, ASEAN + 3 and TPP nations by pharmaceutical group (thousand USD). [file 40064_2016_3200_MOESM4_ESM.docx]

Additional file 4 Changes in Vietnam's pharmaceutical imports from the EU, ASEAN+3 and TPP nations by pharmaceutical group (Unit: thousand USD)

| **Nation** | **HS 3003** | | | **HS 3004** | | | **HS 3005** | | | **HS 3006** | | |
| --- | --- | --- | --- | --- | --- | --- | --- | --- | --- | --- | --- | --- |
|  | **Scenario 1** | **Scenario 2** | **Difference** | **Scenario 1** | **Scenario 2** | **Difference** | **Scenario 1** | **Scenario 2** | **Difference** | **Scenario 1** | **Scenario 2** | **Difference** |
| Australia | 0.00 | 0.00 | 0.00 | -771.112 | 1270.586 | 2041.698 | 0.00 | 0.00 | 0.00 | 0.00 | 0.00 | 0.00 |
| Austria | 0.00 | 0.00 | 0.00 | 1439.034 | 1308.078 | -130.956 | 0.00 | 0.00 | 0.00 | 0.18 | 0.15 | -0.02 |
| Belgium | 0.00 | 0.00 | 0.00 | 1544.21 | 1387.218 | -156.992 | 0.00 | 0.00 | 0.00 | 0.00 | 0.00 | 0.00 |
| Bulgaria | 0.00 | 0.00 | 0.00 | 396.106 | 354.549 | -41.557 | 0.00 | 0.00 | 0.00 | 0.00 | 0.00 | 0.00 |
| Canada | 0.00 | 0.00 | 0.00 | -136.048 | 230.464 | 366.512 | 0.00 | 0.06 | 0.07 | -2.47 | 2.01 | 4.47 |
| Chile | 0.00 | 0.00 | 0.00 | -37.961 | 67.165 | 105.126 | 0.00 | 0.00 | 0.00 | 0.00 | 0.00 | 0.00 |
| China | 0.00 | 0.00 | 0.00 | -831.669 | -418.209 | 413.46 | -29.11 | -52.00 | -22.89 | -1.35 | -1.51 | -0.16 |
| Cyprus | 0.00 | 0.00 | 0.00 | 676.193 | 598.787 | -77.406 | 0.00 | 0.00 | 0.00 | 0.00 | 0.00 | 0.00 |
| Czech | 0.00 | 0.00 | 0.00 | 12.743 | 11.554 | -1.189 | 0.00 | 0.00 | 0.00 | 0.00 | 0.00 | 0.00 |
| Denmark | 0.00 | 0.00 | 0.00 | 37.393 | 34.582 | -2.811 | 1.78 | 1.74 | -0.04 | 0.12 | 0.12 | 0.00 |
| Finland | 0.00 | 0.00 | 0.00 | 16.316 | 14.874 | -1.442 | 1.90 | 1.86 | -0.04 | 0.00 | 0.00 | 0.00 |
| France | 24.92 | 24.92 | 0.00 | 7917.873 | 7160.629 | -757.244 | 298.04 | 286.41 | -11.62 | 233.11 | 205.06 | -28.06 |
| Germany | 0.00 | 0.00 | 0.00 | 6490.424 | 5883.156 | -607.268 | 27.95 | 26.48 | -1.47 | 96.64 | 83.79 | -12.85 |
| Greece | 0.00 | 0.00 | 0.00 | 420.734 | 197.395 | -223.339 | 0.00 | 0.00 | 0.00 | 0.00 | 0.00 | 0.00 |
| Hungary | 0.00 | 0.00 | 0.00 | 769.263 | 704.454 | -64.809 | 0.00 | 0.00 | 0.00 | 1.80 | 1.56 | -0.24 |
| Indonesia | 0.00 | 0.00 | 0.00 | -360.274 | -253.399 | 106.875 | -12.73 | -15.56 | -2.83 | -2.99 | -3.36 | -0.37 |
| Ireland | 0.00 | 0.00 | 0.00 | 1022.523 | 929.468 | -93.055 | 1.25 | 1.23 | -0.03 | 72.52 | 62.87 | -9.65 |
| Italy | 0.00 | 0.00 | 0.00 | 4161.983 | 3748.504 | -413.479 | 4.98 | 4.72 | -0.27 | 19.94 | 17.29 | -2.66 |
| Japan | 0.00 | 0.00 | 0.00 | -470.49 | -342.811 | 127.679 | -7.08 | 23.36 | 30.44 | -9.57 | -2.73 | 6.84 |
| Korea | -2.13 | -2.13 | 0.00 | -2456.225 | -1331.822 | 1124.403 | -17.45 | 75.13 | 92.58 | -3.96 | -4.45 | -0.49 |
| Latvia | 0.00 | 0.00 | 0.00 | 5.705 | 0 | -5.705 | 0.00 | 0.00 | 0.00 | 0.00 | 0.00 | 0.00 |
| Lithuania | 0.00 | 0.00 | 0.00 | 0.25 | 0.228 | -0.022 | 0.00 | 0.00 | 0.00 | 0.00 | 0.00 | 0.00 |
| Luxembourg | 0.00 | 0.00 | 0.00 | 0.00 | 0.00 | 0.00 | 0.00 | 0.00 | 0.00 | 0.00 | 0.00 | 0.00 |
| Malaysia | 0.00 | 0.00 | 0.00 | -238.308 | -261.377 | -23.069 | -0.34 | -0.42 | -0.09 | 0.00 | 0.00 | 0.00 |
| Malta | 0.00 | 0.00 | 0.00 | 15.799 | 14.403 | -1.396 | 0.00 | 0.00 | 0.00 | 0.00 | 0.00 | 0.00 |
| Mexico | 0.00 | 0.00 | 0.00 | -141.358 | 234.746 | 376.104 | -0.01 | 0.21 | 0.22 | 0.00 | 0.00 | 0.00 |
| Netherlands | 0.00 | 0.00 | 0.00 | 490.344 | 444.299 | -46.045 | 3.92 | 3.71 | -0.21 | 0.16 | 0.14 | -0.02 |
| New Zealand | 0.00 | 0.00 | 0.00 | -2.155 | 4.529 | 6.684 | 0.00 | 0.00 | 0.00 | 0.00 | 0.00 | 0.00 |
| Peru | 0.00 | 0.00 | 0.00 | -2.433 | 4.764 | 7.197 | 0.00 | 0.00 | 0.00 | 0.00 | 0.00 | 0.00 |
| Philippines | 0.00 | 0.00 | 0.00 | -131.518 | -129.697 | 1.821 | -0.34 | -0.51 | -0.16 | 0.00 | 0.00 | 0.00 |
| Poland | 0.00 | 0.00 | 0.00 | 796.727 | 709.239 | -87.488 | 0.55 | 0.54 | -0.01 | 0.00 | 0.00 | 0.00 |
| Portugal | 0.00 | 0.00 | 0.00 | 233.661 | 211.736 | -21.925 | 1.84 | 1.78 | -0.06 | 0.00 | 0.00 | 0.00 |
| Romania | 0.00 | 0.00 | 0.00 | 246.6 | 223.61 | -22.99 | 0.00 | 0.00 | 0.00 | 0.00 | 0.00 | 0.00 |
| Singapore | 0.00 | 0.00 | 0.00 | -295.577 | -324.041 | -28.464 | -0.03 | -0.04 | -0.01 | 0.00 | 0.00 | 0.00 |
| Slovak | 0.00 | 0.00 | 0.00 | 4.835 | 4.407 | -0.428 | 0.00 | 0.00 | 0.00 | 0.00 | 0.00 | 0.00 |
| Slovenia | 0.00 | 0.00 | 0.00 | 164.338 | 144.881 | -19.457 | 0.00 | 0.00 | 0.00 | 0.00 | 0.00 | 0.00 |
| Spain | 2.04 | 2.04 | 0.00 | 1341.67 | 1210.89 | -130.78 | 5.76 | 5.52 | -0.24 | 2.22 | 1.92 | -0.30 |
| Sweden | 0.00 | 0.00 | 0.00 | 1029.655 | 938.735 | -90.92 | 0.00 | 0.00 | 0.00 | 0.10 | 0.09 | -0.01 |
| Thailand | -0.84 | -0.84 | 0.00 | -882.133 | -388.074 | 494.059 | -64.44 | -134.90 | -70.46 | -0.08 | 0.09 | 0.17 |
| UK | 8.92 | 8.92 | 0.00 | 4230.824 | 3754.398 | -476.426 | 31.77 | 31.04 | -0.73 | 4.56 | 3.95 | -0.61 |
| US | 0.00 | 0.00 | 0.00 | -1274.979 | 2163.732 | 3438.711 | -7.83 | 137.03 | 144.87 | -47.72 | 38.65 | 86.37 |
| **Total** | 32.92 | 32.92 | 0.00 | 25432.963 | 30516.63 | 5083.667 | 240.38 | 397.39 | 157.01 | 363.21 | 405.63 | 42.43 |

*Source:* Author's calculation from SMART simulation results
